# ISPAT-3D: Spatially Varying Conditional Volumetric Network Estimation for 3D Tumor Imaging

**DOI:** 10.21203/rs.3.rs-9903140/v1

**Published:** 2026-07-03

**Authors:** Sagnik Bhadury, Arvind Rao

**Affiliations:** 1 Department of Computational Medicine and Bioinformatics, University of Michigan, Ann Arbor, MI, USA; 2 Department of Biostatistics, University of Michigan, Ann Arbor, MI, USA; 3 Department of Biomedical Engineering, University of Michigan, Ann Arbor, MI, USA

## Abstract

The spatial organization of the tumor microenvironment shapes immune function and disease progression, yet existing methods for cell-type interaction networks from multiplexed tissue images operate in two dimensions and ignore spatial autocorrelation. We introduce ISPat-3D (Informed Spatially Aware Patterns in 3D), a hierarchical Bayesian framework that recovers spatially varying, zone-specific interaction networks from 3D multiplexed cancer imaging data. The method partitions the tissue volume into tumor intensity zones, fits an anisotropic Gaussian process per cell type and zone with separate lengthscales for the tissue plane and axial direction, decomposes the residuals via multi-study factor analysis, and extracts partial correlation networks from the resulting precision matrices. Simulations demonstrate accurate recovery of shared and zone-specific structure with high power and controlled FDR. We apply ISPat-3D to two 3D serial section multiplexed datasets: the colorectal cancer atlas (CRC1) 3D CyCIF specimen and a HER2-positive ductal breast carcinoma (BC) specimen from a 3D IMC. In CRC1, zone-specific networks reveal a T cell module intensifying with tumor burden, with the dominant regulatory association shifting from CD4^+^↔Treg at intermediate density to CD8^+^↔Treg at maximal density, consistent with cytotoxic suppression at the tumor core. In BC, the shared network shows near-perfect conditional coupling between cancer-associated fibroblasts and the myoepithelial layer, while zone-specific networks reveal CAF↔endothelial co-localisation at intermediate and high burden, consistent with angiogenic remodeling, and a B cell↔CAF association confined to high-density zones, consistent with tertiary lymphoid structure formation. Across both tumors, ISPat-3D identifies volumetric spatial conditional interactions not recoverable from 2D sections. The code for ISPAT-3D is available at https://github.com/sagnikbhadury/ISPAT-3D.

## Introduction

The tumor microenvironment (TME) is a spatially organized ecosystem in which malignant, immune, and stromal cells interact in a manner that is inextricably linked to tumor progression, immune evasion, and therapeutic response^1^. A defining feature of solid tumors is that these interactions are not uniform across the tissue. Instead, they vary systematically across spatial gradients defined by tumor cell density, proximity to the invasive margin, and local immune infiltration status. Characterizing these spatially varying interaction patterns at the level of individual cell types, rather than averaging over the whole tumor, is increasingly recognized as essential for understanding the mechanisms of immunosuppression and for identifying spatially resolved biomarkers of prognosis and treatment response^2^.

Highly multiplexed tissue imaging technologies have made this goal tractable. Platforms including cyclic immunofluorescence (CyCIF)^3^, imaging mass cytometry (IMC)^4^, multiplexed ion beam imaging (MIBI)^5^, and co-detection by indexing (CODEX)^6^ now enable simultaneous quantification of tens to over one hundred protein markers at single-cell resolution in intact tissue sections while preserving spatial context. Applied to cancer specimens, these technologies have fundamentally changed the scope of spatial biology. In triple-negative breast cancer, MIBI imaging of 36 proteins revealed that the spatial organization of the tumor-immune interface – whether immune cells were mixed within or compartmentalized away from tumor cells – stratified patients by survival independently of immune cell abundance^5^. In colorectal cancer, CODEX profiling of lymph node metastases identified multicellular neighborhoods that coordinate antitumoral immunity at the invasive front^7^. In pancreatic cancer,^8^ identified that spatially, multiple immune related ligand receptor interactions differ across precancerous lesions and pancreatic ductal adenocarcinoma patient groups given the tumor trajectory. Across tumor types, these studies established that spatial architecture, not just cellular composition, determines immune function.

The analysis of multiplexed imaging data has to date been almost entirely two-dimensional, treating each tissue section as an independent spatial domain. However, solid tumors are inherently three-dimensional objects, and tissue sections represent thin planar cuts through a volumetric structure whose cellular architecture varies substantially along the depth axis. The relevance of this limitation has been demonstrated directly:^9^ developed 3D imaging mass cytometry by stacking serial FFPE sections and showed that measurements of cellular proximity and spatial neighborhood composition differed materially between 3D and 2D representations of the same tumor, with 2D imaging overestimating the distance between cell clusters and blood vessels.^10^ extended this approach to colorectal cancer at a scale of 2 × 10^8^ cells across 25 serial sections, generating what remains the most detailed 3D single-cell atlas of a human tumor, and demonstrated that mesoscale tumor features including long-range oncogene expression gradients and the architecture of the PD1:PDL1 immune checkpoint interface can only be detected in three-dimensional data.^11^ utilized 3D mapping on pancreatic cancer and revealed that inflammation around individual precancers is highly heterogeneous, with immune hotspots and cold spots interchanging over tens of microns. These findings collectively establish that 3D multiplexed imaging is not merely a technical increment but a qualitatively different window into tumor biology.

Despite these advances in data acquisition, the statistical methodology for analyzing 3D multiplexed imaging data remains underdeveloped. Existing spatial analysis tools including co-occurrence statistics, cross-correlation functions, nearest-neighbor enrichment scores, and cellular neighborhood methods were designed for two-dimensional point process data and make no use of the depth axis. They also do not account for spatial autocorrelation when estimating cell-type associations, which is a substantive confound in tissues where globally structured spatial variation in cell density inflates pairwise co-occurrence measures. More fundamentally, existing methods produce a single summary network for each spatial region, discarding the spatial heterogeneity that motivates 3D analysis in the first place. They cannot recover how the conditional dependence structure among cell types shifts along the tumor density gradient, which is precisely the question of biological interest.

Statistical methods for spatial genomics have made considerable progress in handling spatial autocorrelation, primarily in the context of spatial transcriptomics. Gaussian process (GP) regression has been used to identify spatially variable genes by decomposing expression variation into a spatially structured component and a residual^8,12,13^. These approaches model the smooth spatial trend as a nuisance and extract the signal of interest from the residual. This is the conceptual precedent for the GP deconfounding stage in ISPat-3D, though our setting differs substantially: we are not identifying spatially variable markers in isolation but rather recovering the joint residual covariance structure across multiple cell types simultaneously, in three dimensions, and stratified by tumor zones. For cell-cell interaction inference from spatial transcriptomics, methods based on ligand-receptor databases^14^ and optimal transport^15^ have been proposed, but these operate on categorical proximity and do not model continuous spatial intensity surfaces or partial correlation networks.

The multi-study factor analysis (MSFA) framework of^16^, extended to the high-dimensional Bayesian setting by^17^, provides the second key statistical component of ISPat-3D. MSFA decomposes a collection of covariance matrices from multiple studies into a component shared across all studies and study-specific residual components, using a latent factor structure. In our setting, the five tumor density zones defined by local tumor cell density play the role of studies, and the shared and zone-specific loading matrices recover the global and zone-varying cell-type interaction networks respectively. Variational inference algorithms for MSFA that substantially reduce computational cost relative to Markov chain Monte Carlo sampling have recently been developed^18^, and we rely on these to make inference tractable at the scale of 3D multiplexed data. On the other hand Informed Spatially Aware Patterns for Multiplexed Immunofluorescence Data^8^ is the building block of this manuscript. ISPAT models cell-cell interaction profile varying over tumor trajectory using a Gaussian process mixed effect model through conditional independence structure through multi-study factor analytic form and is applied to 2D multiplexed pancreatic cancer data.

In this manuscript, we introduce ISPat-3D (Informed Spatially Aware Patterns in Three Dimensions), a hierarchical Bayesian framework that operates directly on three-dimensional multiplexed imaging data to produce spatially varying, zone-specific cell-type interaction networks. The method proceeds in three stages. First, an anisotropic Gaussian process is fit to each cell-type intensity surface within each tumor density zone, using separate lengthscale parameters for the tissue plane and the depth axis to account for the anisotropic resolution structure of serial section imaging. The spatially adjusted residuals from this stage are then passed to MSFA, which decomposes the cross-cell-type covariance into a network component shared across all zones and zone-specific network perturbations. From the undirected network structures we could focus on individual bidirectional interaction patterns of two cell types accounting for rest of the cell types to understand the spatial interactions among cell types. Finally, partial correlation networks are extracted from the resulting precision matrices to represent conditional co-localisation structure among cell types at each level of tumor density. The interpretation of the interaction patterns obtained through the analysis are volumetric, conditional, bidirectional and literature addressing this problem is sparse.

**Figure 1.**
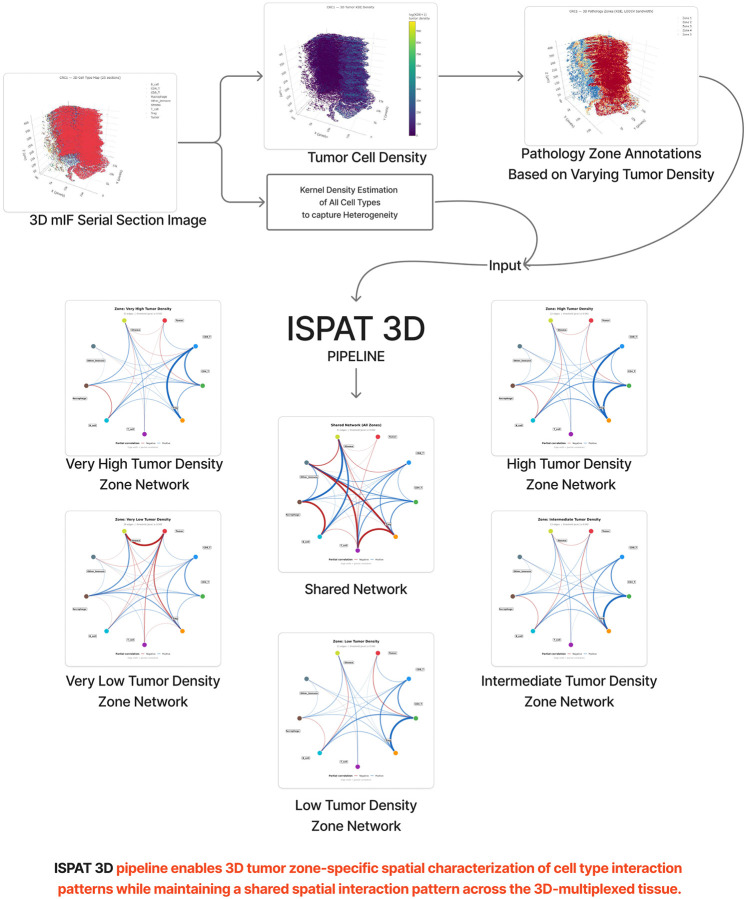
Schematic overview of the ISPat-3D framework. The pipeline transforms histopathological mIF images into point pattern representations of cellular distributions in the tissue, delineates tumor subregions (zones) based on heterogeneity of tumor cells, obtains marginal spatial kernel density estimates, and identifies tumor subregion/zone-based cellular heterogeneity patterns as well as shared cellular patterns. Optional integration of domain knowledge of cellular interactions into the pipeline can be used when needed.

We first apply ISPat-3D to the CRC1 specimen from the 3D colorectal cancer atlas of^10^, a poorly differentiated stage IIIB BRAF^V600E^ adenocarcinoma with high microsatellite instability and complex invasive margin histomorphology. For generalizability purpose, we then apply it to the main specimen of breast carcinoma 3D imaging mass cytometry data from^9^. To our knowledge, ISPat-3D is the first statistical method designed to recover spatially varying, zone-specific interaction networks from three-dimensional cancer imaging data. We evaluate its performance through a simulation study across multiple kernel specifications, count configurations, and zone size settings, and demonstrate that the method recovers both shared and zone-specific network structure with high accuracy and practical computational cost. In the CRC1 application, ISPat-3D reveals a shared macrophage↔stroma co-localisation signal and a coordinated T cell module that intensifies along the tumor density gradient, with a shift toward cytotoxic T cell↔regulatory T cell proximity at maximal tumor density consistent with active immunosuppression at the tumor core. In the breast carcinoma application, ISPat-3D uncovers a near-perfect conditional coupling between cancer-associated fibroblasts and the myoepithelial layer at low tumor density, a CAF↔endothelial co-localisation that emerges specifically at intermediate and high tumor burden and reflects density-dependent angiogenic remodeling, and a B cell↔CAF association confined to high-density zones that is consistent with tertiary lymphoid structure formation in regions of elevated antigen load. We also show that across both tumors, the zone-specific networks reveal interaction structure that is invisible to any single two-dimensional serial section and that cannot be recovered by existing 2D spatial methods. We present the schematic overview of the pipeline in Figure 1.

## Results

### Simulation Study

#### Data Generating Mechanism

We designed a simulation study to evaluate the ability of ISPat-3D to recover shared and zone-specific interaction networks under controlled conditions. Synthetic data were generated using a hierarchical model that mirrors the assumed structure of the ISPat-3D pipeline, incorporating anisotropic three-dimensional spatial dependence, multi-study factor structure across zones, and realistic cluster size configurations.

For each replication, spatial locations for Q=3 zones were drawn as follows. The (x,y) coordinates for cells in zone q were sampled from a bivariate Gaussian centered at (3q,3q) with standard deviation 1.5, and the z-coordinate was drawn independently from Uniform(0, 1). Zones are thus spatially separated in the tissue plane while sharing a common z-range, reflecting the confocal section structure of multiplexed data.

The multi-study factor structure was specified with K=⌈2logG⌉ shared latent factors, matching the factor count used at estimation time. Shared loadings $𝚽\in R^{G\times K}$ were drawn entry-wise from 𝒩(0,1). Zone-specific loadings ${𝚲}_{q}\in R^{G\times K_{s}}$ were similarly drawn, with the number of zone-specific factors $K_{s}$ sampled uniformly from {2,…,K−1} with replacement across zones. Idiosyncratic variances were drawn from Uniform(0.1, 1). The true zone-level covariance matrices were then ${𝚺}_{q}=𝚽{𝚽}^{⊤}+{𝚲}_{q}{𝚲}_{q}^{⊤}+{𝚿}_{q}$ from which multivariate Gaussian cell profiles $Z_{q}\in R^{N_{q}\times G}$ were sampled row-wise, with each row representing the network signal for one cell.

Anisotropic spatial random effects $B_{g}\in R^{N_{q}}$ were drawn per cell per zone from a zero-mean multivariate Gaussian with covariance $\sigma_{S,gq}^{2}K_{q}$, where $\sigma_{S,gq}^{2}\sim \text{Uniform}(0.16,0.36)$ and $K_{q}$ is the correlation matrix under either the Matérn-3/2 or RBF kernel. Both kernels use separate lengthscales $\ell_{S}$ and $\ell_{Z}$ for the (x,y) plane and the z-axis respectively, computed as the median pairwise distance among cells within zone q along each axis. The final observed cell matrix Y was generated as

(1) $$f_{q}^{g}\left(s_{j}\right)=\beta_{gq}+B_{g}\left(s_{j}\right)+\zeta_{g}^{q}\left(s_{j}\right)+\varepsilon_{j},\;\varepsilon_{j}\sim 𝒩\left(0,\sigma_{\varepsilon }^{2}\right),$$

where $\beta_{gq}\sim \text{Uniform}(1,3)$ are cell and zone-specific intercepts, $\zeta_{g}^{q}\left(s_{j}\right)$ is the g-th element of the j-th row of $Z_{q}$, and $\sigma_{\varepsilon }=0.5$. The spatial nuisance $B_{g}$ and network signal $\zeta_{g}^{q}$ are thus additive and independent by construction, providing a clean ground truth against which to assess Stage 1 spatial deconfounding and Stage 2 network recovery.

#### Simulation Design

We evaluated ISPat-3D across a factorial combination of settings. The number of cells was varied over G∈{15,20,25}. Six cluster size configurations were considered, spanning balanced and unbalanced allocations across the three zones: (300, 300, 300), (250, 250, 250), (250, 300, 300), (250, 250, 300), (300, 250, 250), and (300, 300, 250). Both kernel choices (Matérn-3/2 and RBF) were evaluated. All results were averaged over 10 independent replications.

The ground truth shared network was defined as ${\hat{W}}_{\text{shared}}^{\text{true}}=𝚽{𝚽}^{⊤}$, targeting the low-rank shared covariance recovered by the MSFA layer. Zone-specific networks were defined as ${\hat{W}}_{q}^{\text{true}}=𝚽{𝚽}^{⊤}+{𝚲}_{q}{𝚲}_{q}^{⊤}+{𝚿}_{q}$, where ${𝚿}_{q}$ is the zone-specific idiosyncratic variance, consistent with the generative covariance structure in the Network Construction section of Methods. Ground truth matrices were extracted within each replicate independently to ensure alignment between the simulation draws and the corresponding estimated networks.

#### Evaluation Metrics

Three complementary metrics were used to assess recovery performance. The RV coefficient^19^ measures similarity between two positive semidefinite matrices and takes values in [0, 1], with RV = 1 indicating perfect structural agreement. It was computed between the true and estimated network matrices for both the shared network and each of the three zone-specific networks. For edge detection, both the true and estimated networks were binarized using an absolute entry threshold of 0.1: a true edge was declared wherever the true network had a nonzero off-diagonal entry, and an estimated edge wherever $\left|{\hat{W}}_{gg^{\prime }}\right|>0.1$. Statistical power (true positive rate) and false discovery rate (FDR) were then computed from the resulting confusion matrix. Computation time in seconds was recorded as a practical scalability metric.

#### Results of Simulation Study

##### Network recovery accuracy (RV coefficient).

**Figure 2.**
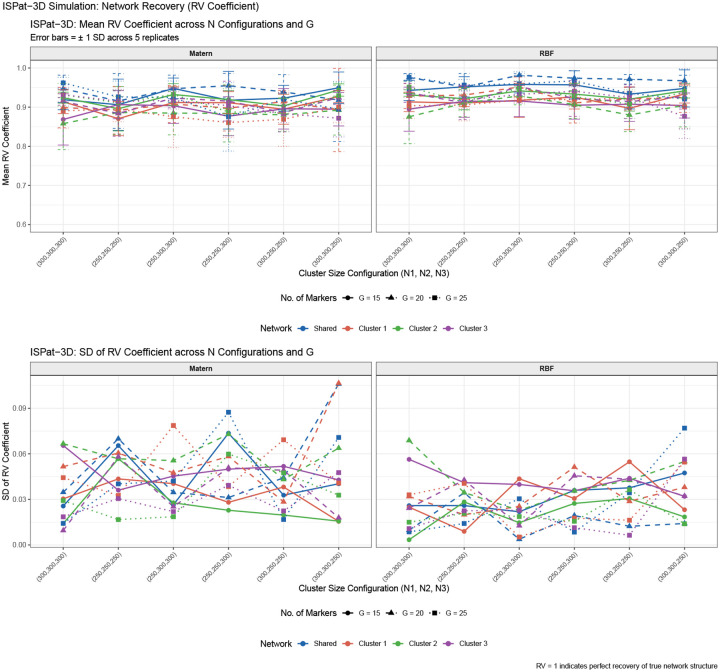
Simulation results for ISPat-3D: mean RV coefficient (top) and its standard deviation across 5 replicates (bottom) as a function of cluster size configuration and number of cells G, faceted by kernel. Error bars represent ±1 SD. RV = 1 indicates perfect recovery of the true network structure.

Figure 2 shows mean RV coefficients across all cluster size configurations, cell counts, and kernels. ISPat-3D achieves consistently high RV values throughout, with mean RV ranging from approximately 0.65 to 1.0 across all settings. The shared network is recovered with the highest fidelity, which is expected since the shared component pools information across all three zones. Zone-specific networks are recovered with slightly lower but still strong RV values, reflecting the additional difficulty of disentangling zone-level deviations from the shared structure. Performance is largely stable across the six cluster size configurations, indicating robustness to moderate imbalance in zone sizes. The Matérn-3/2 and RBF kernels perform comparably in terms of mean RV, though Matérn-3/2 exhibits marginally lower variability across replicates. Increasing G from 15 to 25 does not systematically degrade recovery, suggesting the MSFA layer scales appropriately with the number of cells. The SD of the RV coefficient remains below 0.10 across all settings, confirming stable estimation across replications.

##### Edge detection: power and false discovery rate.

**Figure 3.**
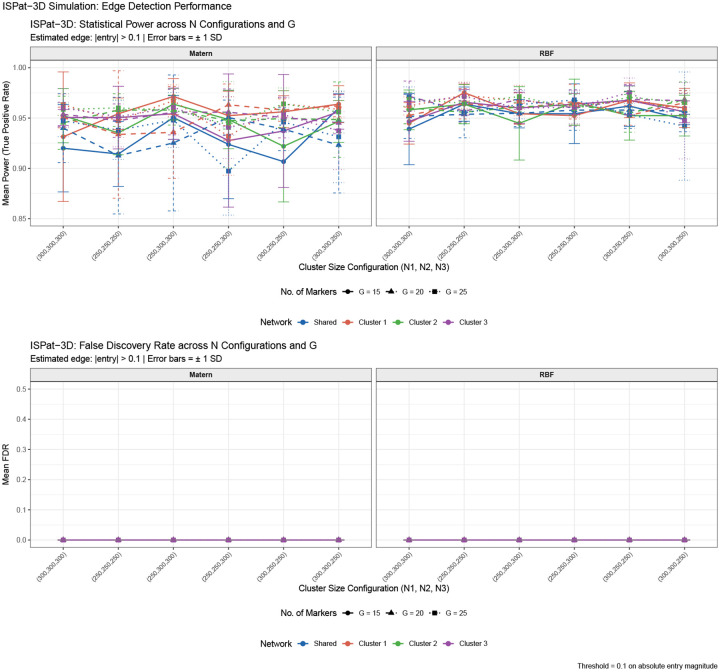
Simulation results for ISPat-3D: statistical power (top) and false discovery rate (bottom) for edge detection as a function of cluster size configuration and number of cells G, faceted by kernel. An edge is declared in the estimated network wherever the absolute entry exceeds 0.1. Error bars represent ±1 SD across 10 replicates.

Figure 3 presents statistical power and FDR for edge detection across all settings. ISPat-3D achieves high power throughout, consistently above 0.85 across all configurations and kernels, with mean power approaching 1.0 under balanced and larger cluster configurations. Power is uniformly strong for both shared and zone-specific networks, with no systematic degradation as G increases. FDR behavior is more variable across the two network types. The shared network maintains low FDR (near zero) in all settings, while zone-specific networks exhibit moderate FDR ranging up to approximately 0.1 at the smallest cluster sizes, with values decreasing as $N_{c}$ increases, consistent with improved estimation precision at larger sample sizes. This pattern reflects the expected operating characteristic of a fixed threshold under varying signal-to-noise conditions: the threshold of 0.1 is sufficiently conservative to control spurious edge declarations in the shared network, where pooling across zones yields high effective sample size, but is somewhat liberal for zone-specific networks when $N_{c}$ is small.

##### Computation time.

**Figure 4.**
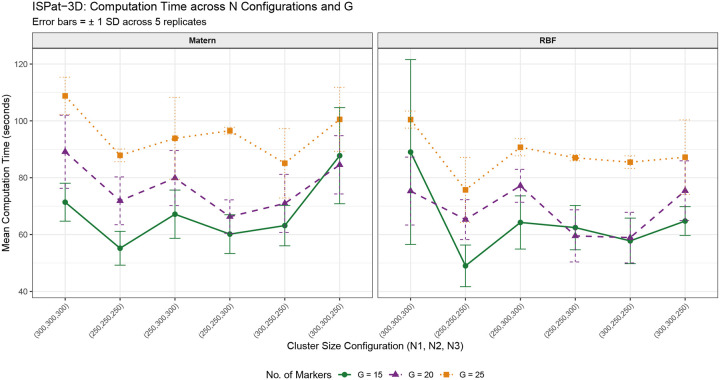
Simulation results for ISPat-3D: mean computation time in seconds as a function of cluster size configuration and number of cells G, faceted by kernel. Error bars represent ±1 SD across 10 replicates.

Figure 4 shows mean computation time as a function of cluster size configuration and cell count, faceted by kernel. Computation time scales roughly linearly with total cell count and with G, consistent with the per-cell parallelized GP fitting underlying the spatial deconfounding step. The Matérn-3/2 kernel incurs a modest additional cost relative to RBF, attributable to the positive definiteness check and nearest-PD projection applied to the Matérn-3/2 covariance matrix. Across all settings, computation times range from approximately 40 to 120 seconds, confirming that ISPat-3D is computationally tractable. We emphasize that computational time increases especially due to the optimization step involved but at the same time network estimation accuracy increases, with the number of spatial points in consideration for analysis. We have run this method using large memory high performance clusters at the scale of CyCIF and IMC datasets considered in this manuscript.

### Colorectal Cancer Data Description

We applied ISPat-3D to the colorectal cancer (CRC) cyclic immunofluorescence (CyCIF) dataset released by^10^. The dataset comprises a single specimen subjected to full 3D serial section imaging (CRC1). Since ISPat-3D requires a true 3D spatial structure – namely, consistent global XY coordinates across registered serial sections and a physical Z axis – CRC1 satisfies the structural requirements of the method.

CRC1 is a poorly differentiated stage IIIB BRAF^V600E^ colorectal adenocarcinoma with high microsatellite instability and complex histomorphology. The specimen was serially sectioned into 106 FFPE slices of 4μm thickness, from which 25 CyCIF sections were selected at non-consecutive physical indices to maximize the total reconstructed depth along the Z-axis while skipping interleaved H&E sections. Each section was assigned a physical depth coordinate $Z_{\mu \text{m}}=s\times 4$, where s is the section index, yielding a total reconstructed depth spanning 8 to 424μm. Whole-slide CyCIF imaging was performed using 102 antibodies covering epithelial, immune, and stromal lineage markers as well as markers of cell cycle state, signaling activity, and immune checkpoint expression^10^. Images were stitched, registered across serial sections, and segmented using the MCMICRO pipeline^20^, yielding globally consistent tissue-level XY coordinates $\left(X_{s},Y_{s}\right)$ for each segmented cell that are directly comparable across all 25 Z-sections.

### 3D CyCIF Colorectal Cancer Data Analysis

#### Spatial Sampling and Kernel Density Estimation

To this end, the whole slide 3D image cells were stratified by tumor intensity zone, defined as five ordered categories of local tumor cell density: Very Low, Low, Intermediate, High, and Very High capturing the tumor cell’s heterogeneity gradient precisely. Due to the volume of the data with billions of cells present, for computational constraint and methodological boundary, within each zone, we drew three independent spatially stratified random subsamples of 50000,100000, and 150000 cells, where stratification was performed on a grid of the xyz-coordinates to preserve spatial coverage across the tissue section. For each subsample and each cell type, we estimated a continuous spatial intensity surface using kernel density estimation (KDE), evaluated at the observed cell locations. The resulting KDE surfaces were log-transformed to stabilize the dynamic range prior to downstream modeling.

#### ISPat-3D Pipeline

The ISPat-3D framework was applied to jointly model the spatial co-distribution of nine cell types: Tumor, CD8^+^ T cells (CD8_T), CD4^+^ T cells (CD4_T), regulatory T cells (Treg), pan-T cells (T_cell), B cells, Macrophages, Other immune cells, and Stroma. For each cell type and each zone, a Gaussian process (GP) regression was fit to the log-transformed KDE surface using a Matérn kernel with separate spatial and axial lengthscale parameters, both optimized via L-BFGS-B marginal likelihood. The spatially adjusted residual for each cell type was obtained by subtracting the estimated spatial trend from the observed surface. MSFA decomposed the residual matrix across zones into a shared factor loading matrix 𝚽 and zone-specific loading matrices ${𝚲}_{q}$, estimated via coordinate ascent variational inference as described previously. From these, a shared covariance network ${𝚺}_{\text{shared}}$ and zone-specific covariance networks ${𝚺}_{q}$ were constructed, each of dimension G×G where G=9.

#### Aggregation Across Subsampling Replicates

To reduce dependence on any single subsample, partial correlation networks were estimated separately for each of the three subsampling replicates (50000, 100000, and 150000 cells per zone) and then combined. For each replicate, partial correlations were obtained from the covariance network via precision matrix inversion: given $𝚯={𝚺}^{-1}$, the partial correlation between cell types i and j is

$$\rho_{ij}=-\frac{\Theta_{ij}}{\sqrt{\Theta_{ii}\Theta_{jj}}}.$$

**Figure 5.**
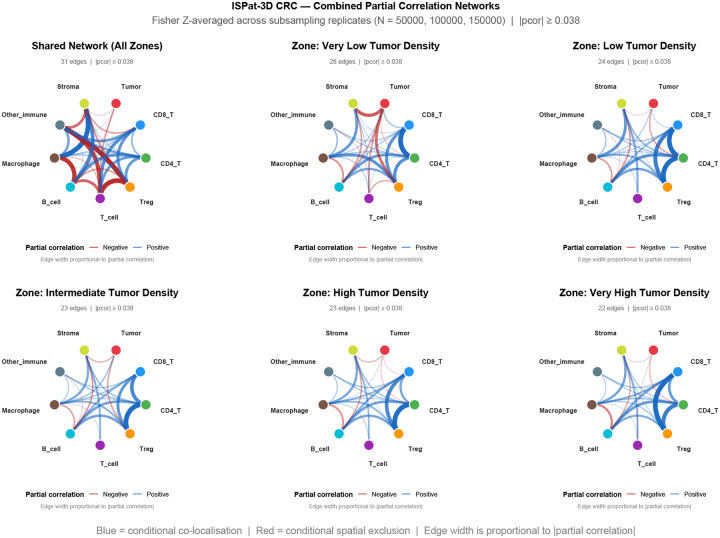
ISPat-3D partial correlation networks for CRC1 across tumor intensity zones. Each panel displays the Fisher Z-averaged partial correlation network estimated from three independent spatially stratified subsamples (50000, 100000, and 150000 cells per zone). Nodes represent the nine cell types profiled. Edges are shown for pairs with $|\overset{ˆ}{\rho }|\geq 0.038$ (30th percentile of all absolute partial correlations). Blue edges denote positive partial correlations, indicating conditional spatial co-localisation; red edges denote negative partial correlations, indicating conditional spatial exclusion. Edge width is proportional to $\left|\overset{ˆ}{\rho }\right|$. The shared network (top left) reflects associations common to all zones; the remaining five panels show zone-specific networks ordered by increasing tumor cell density from Very Low (top center) to Very High (bottom right).

The three replicate partial correlation matrices were then aggregated using the Fisher Z-transform. Each element $\rho_{ij}^{\left(k\right)}$ was mapped to $z_{ij}^{\left(k\right)}={\text{tanh}}^{-1}\left(\rho_{ij}^{\left(k\right)}\right)$, averaged across replicates, and back-transformed: ${\overset{ˆ}{\rho }}_{ij}=\text{tanh}\left({\overset{‾}{z}}_{ij}\right)$. This approach is statistically preferable to direct averaging because partial correlations are bounded in [−1, 1] and their sampling distribution is skewed near the boundary, whereas the Fisher Z-transform maps them to an approximately normal, unbounded scale on which averaging is valid. Edges with $\left|{\overset{ˆ}{\rho }}_{ij}\right|$ below the 30th percentile of all off-diagonal absolute partial correlations were suppressed to aid visual clarity. The resulting six networks, one shared and five zone-specific, are displayed in Figure 5.

#### Cell-Type Interaction Networks Across Tumor Density Zones

ISPat-3D was applied to the CRC1 specimen to recover a shared interaction network and five zone-specific networks stratified by local tumor cell density. The conditional associations per network are displayed as partial correlation networks in Figure 5. We describe each network in turn noting that these interaction patterns are recovered as conditional independence structures that vary over tumor trajectory keeping the volumetric feature of the 3D data in account. Given the formulation of ISPat-3D, we can now uncover the ligand receptor interaction graphs which are not captured via any method built for 2D image analysis.

##### Shared network.

The shared network captures conditional associations that are consistent across all five tumor density zones and therefore reflects structural features of the tumor microenvironment that are not specific to any local tumor burden regime. The dominant edge is a strong positive partial correlation between Macrophage and Stroma $\left(\overset{ˆ}{\rho }=0.956\right)$, indicating that macrophage spatial density co-varies with stromal density even after accounting for all other cell types and removing large-scale spatial trends via the Gaussian process stage. This pattern is consistent with the well-established localization of tumor-associated macrophages within stromal compartments in colorectal cancer^10^, but the magnitude and zone-independence of this conditional association have not previously been quantified as a network feature in three-dimensional imaging data. The second and third strongest edges are negative: Other_immune cells are negatively associated with both Treg $\left(\overset{ˆ}{\rho }=-0.953\right)$ and T_cell $\left(\overset{ˆ}{\rho }=-0.803\right)$, and T_cell is negatively associated with Treg $\left(\overset{ˆ}{\rho }=-0.927\right)$. These large negative partial correlations suggest spatial exclusion between the conventional T cell compartment and regulatory immune populations after conditioning on the full cell-type composition, a pattern that is not captured by simple co-occurrence or nearest-neighbor proximity analyses. Notably,^10^ described Treg co-localization with CD8+ cytotoxic T cells at the invasive margin as a hallmark of immune suppression in this tumor, but that analysis was based on marginal spatial proximity rather than conditional network estimation. The negative partial correlations we observe here suggest that, when the full immunological context of each region is held fixed, Tregs and conventional T cells occupy mutually exclusive spatial niches at a tissue-wide level, which is not recoverable from 2D pairwise proximity analysis and is obscured in any single histological section by the section-to-section variability in immune infiltration depth.

Two additional shared edges extend beyond what^10^ characterized: a positive association between B_cell and Stroma $\left(\overset{ˆ}{\rho }=0.706\right)$ and a negative association between B_cell and Macrophage $\left(\overset{ˆ}{\rho }=-0.868\right)$. The B cell compartment in CRC1 was noted in the original study primarily in the context of tertiary lymphoid structures (TLS), but the conditional spatial coupling of B cells to the stromal scaffold and their conditional exclusion from macrophage-dense regions were not modeled. These edges suggest that the stromal architecture serves as the organizing substrate for B cell positioning in this tumor while simultaneously being the environment from which macrophages exclude B cells, possibly because macrophage-dense stromal niches are polarized toward an immunosuppressive rather than a lymphoid-organizing function.

##### Very Low tumor density zone.

In regions of minimal tumor burden, the T cell module is already structurally present: CD4_T and CD8_T cells are positively associated $\left(\overset{ˆ}{\rho }=0.399\right)$, and both CD4_T $\left(\overset{ˆ}{\rho }=0.273\right)$ and CD8_T $\left(\overset{ˆ}{\rho }=0.256\right)$ are positively associated with Treg. The direction of the CD4_T↔Treg and CD8_T↔Treg edges in this zone is positive, in contrast to the negative Treg edges observed in the shared network. This is not a contradiction but a zone-specific deviation from the global pattern: in very low density regions, the immune infiltrate is sparse and heterogeneous, and the few Tregs present tend to co-localize with other T cells rather than exclude them, consistent with early immune priming in a low-antigen environment. Stroma is negatively associated with Tumor $\left(\overset{ˆ}{\rho }=-0.271\right)$ and Treg is also negatively associated with Tumor $\left(\overset{ˆ}{\rho }=-0.229\right)$, suggesting that in low-burden regions, tumor cells and both stromal and regulatory immune cells occupy spatially distinct compartments. This stroma↔tumor negative partial correlation is a three-dimensional observation: in any single 2D section at very low tumor density, tumor nests are scattered and the stroma-tumor boundary is poorly defined, making directional conditional exclusion invisible without integrating information across the z-axis.

##### Low tumor density zone.

The Low zone network is dominated by the T cell regulatory axis. CD4_T↔Treg $\left(\overset{ˆ}{\rho }=0.345\right)$ and CD4_T↔CD8_T $\left(\overset{ˆ}{\rho }=0.340\right)$ are the two strongest edges, with CD8_T↔Treg closely following $\left(\overset{ˆ}{\rho }=0.287\right)$. The B_cell↔CD4_T positive association $\left(\overset{ˆ}{\rho }=0.221\right)$ appears here for the first time and will persist across all subsequent zones, suggesting a stable structural link between the B cell and CD4 T cell compartments that is independent of local tumor burden.^10^ characterized B cells primarily in the context of TLS composition but did not quantify or model the conditional spatial dependence between B cells and CD4 T cells as a recurring network feature. The Macrophage↔Stroma positive edge $\left(\overset{ˆ}{\rho }=0.209\right)$ re-emerges at the zone level, reinforcing the shared network finding. A modest negative association between B_cell and Stroma $\left(\overset{ˆ}{\rho }=-0.114\right)$ and between B_cell and Macrophage $\left(\overset{ˆ}{\rho }=-0.114\right)$ also appears, again consistent with the shared network structure.

##### Intermediate tumor density zone.

The Intermediate zone network retains the same top three edges as the Low zone (CD4_T↔Treg, $\overset{ˆ}{\rho }=0.387$; CD4_T↔CD8_T, $\overset{ˆ}{\rho }=0.290$; CD8_T↔Treg, $\overset{ˆ}{\rho }=0.237$), with the magnitude of the CD4_T↔Treg edge increasing relative to the Low zone, indicating that regulatory T cell spatial coupling to the helper T cell compartment strengthens as tumor burden grows. B_cell↔CD4_T $\left(\overset{ˆ}{\rho }=0.196\right)$ and B_cell↔Treg $\left(\overset{ˆ}{\rho }=0.196\right)$ maintain similar effect sizes, consistent with a B cell compartment that is structurally linked to the regulatory immune niche across intermediate density regions. Two additional edges of biological interest appear in this zone: a negative association between Treg and Tumor $\left(\overset{ˆ}{\rho }=-0.111\right)$ and a positive association between Macrophage and Tumor $\left(\overset{ˆ}{\rho }=0.104\right)$. These opposing signs suggest that at intermediate tumor density, macrophages become more integrated into the tumor-adjacent space while Tregs remain spatially separated from tumor nests, a divergence in the spatial organization of the immunosuppressive machinery that would not be visible in a 2D analysis restricted to a single section because any given section samples the tumor↔immune interface at an arbitrary z-plane.

##### High tumor density zone.

The High zone network shows a consolidation of the T cell regulatory axis alongside the emergence of new B cell and macrophage interactions. CD4_T↔Treg remains the dominant edge $\left(\overset{ˆ}{\rho }=0.389\right)$, with CD8_T↔Treg $\left(\overset{ˆ}{\rho }=0.295\right)$ now higher than CD4_T↔CD8_T $\left(\overset{ˆ}{\rho }=0.187\right)$, signaling a relative shift in which cytotoxic T cell proximity to Tregs becomes more prominent as tumor burden increases. B_cell↔CD4_T $\left(\overset{ˆ}{\rho }=0.204\right)$ and B_cell↔Treg $\left(\overset{ˆ}{\rho }=0.189\right)$ remain stable. The CD8_T↔Macrophage positive edge $\left(\overset{ˆ}{\rho }=0.141\right)$ appears in this zone and the Very High zone but is absent in lower-burden zones, consistent with macrophage recruitment into tumor-dense regions where cytotoxic T cells are also present, a spatial juxtaposition that has been implicated in T cell exhaustion through myeloid checkpoint ligand expression^10^. The negative B_cell↔Macrophage edge $\left(\overset{ˆ}{\rho }=-0.144\right)$ and B_cell↔Stroma edge $\left(\overset{ˆ}{\rho }=-0.111\right)$ persist, supporting the interpretation that macrophage-dense stromal niches in high-burden regions are structurally incompatible with B cell localization.

##### Very High tumor density zone.

The Very High zone network inverts the relative dominance of the two Treg edges that characterized earlier zones: CD8_T↔Treg is now the strongest association $\left(\overset{ˆ}{\rho }=0.373\right)$, surpassing CD4_T↔Treg $\left(\overset{ˆ}{\rho }=0.316\right)$. This shift in the rank ordering of Treg connectivity from CD4-dominated in low-burden zones to CD8-dominated in the highest-burden zone is a gradient-resolved observation that is only accessible through the 3D density zonation approach. In two-dimensional data, Treg co-localization with CD8 T cells is detectable at the invasive margin^10^, but the systematic strengthening of this edge with increasing tumor density cannot be established from a single or even a few 2D sections because local tumor density estimation from a flat image does not reliably reflect volumetric tumor burden. The CD4_T↔Macrophage positive edge $\left(\overset{ˆ}{\rho }=0.111\right)$ also appears exclusively in this zone and is not present at any lower density level, suggesting that helper T cell and macrophage co-localization is a feature of the most tumor-dense microenvironment rather than a general property of the TME. Stroma↔T_cell $\left(\overset{ˆ}{\rho }=0.145\right)$ persists from the Low through Very High zones, a consistent but secondary edge that likely reflects the spatial organization of T cells along stromal channels that permeate the tumor mass.

#### 3D-Specific Volumetric Interactions in CRC1

**Figure 6.**
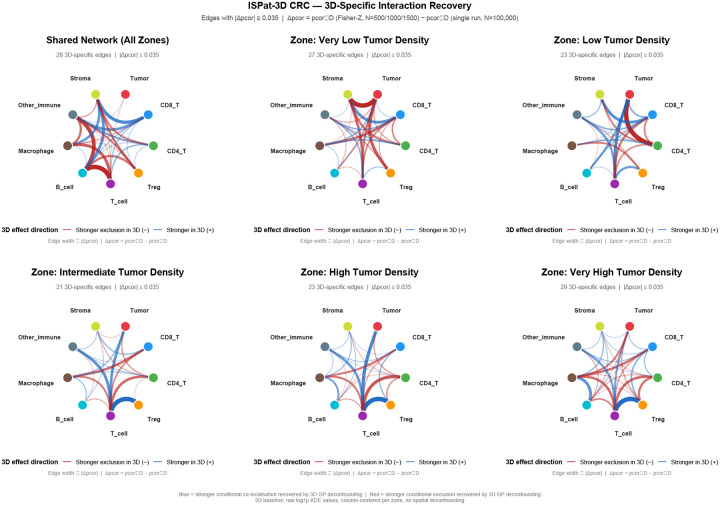
3D-specific interaction networks across tumor density zones in CRC1. Each panel displays edges where $\left|\Delta \rho_{ij}\right|\geq 0.035$, defined as $\Delta \rho_{ij}=\rho_{ij}^{3\text{D}}-\rho_{ij}^{2\text{D}}$. Blue edges indicate conditional co-localisations amplified by 3D GP deconfounding; red edges indicate conditional spatial exclusions amplified by 3D deconfounding. Edge width is proportional to $\left|\Delta \rho_{ij}\right|$. The 2D baseline was fit on raw log-transformed KDE values column-centered per zone with 2D spatial deconfounding.

To evaluate the contribution of three-dimensional spatial deconfounding to network recovery, we applied a 2D baseline variant of ISPat-3D to the CRC1 dataset. This 2D baseline was fit on each serial section across tumor density zones and then combined together with the Fisher Z-transform approach described previously. Partial correlation networks from the 3D and 2D analyses were then compared at the edge level via delta scores defined as $\Delta \rho_{ij}=\rho_{ij}^{3\text{D}}-\rho_{ij}^{2\text{D}}$ for each cell type pair (i,j). Edges were flagged as 3D-specific when $\left|\Delta \rho_{ij}\right|$ exceeded the 30th percentile of all pairwise deltas pooled across networks. A positive delta indicates that the 3D model recovers a stronger conditional co-localisation between two cell types relative to the 2D baseline, meaning that accounting for the vertical tissue axis reveals a spatial affinity that is otherwise obscured by section-level confounding. Conversely, a negative delta indicates that the 3D model recovers a stronger conditional spatial exclusion, meaning that two cell types that appear co-occurring or neutrally distributed in a 2D cross-section are in fact spatially segregated along the depth axis of the tissue. The resulting 3D-specific interaction networks are displayed across all five tumor density zones and the shared network in Figure 6.

In the shared network spanning all zones, the three dominant 3D-specific edges were B cell ↔ T cell (Δρ=−0.987), reflecting a strong conditional exclusion recovered exclusively under 3D deconfounding that is completely masked in the 2D analysis; B cell ↔ Other immune (Δρ=−0.805), indicating a similar pattern of apparent co-occurrence in 2D that resolves into spatial segregation once the vertical tissue axis is modeled; and CD8^+^ T cell ↔ Stroma (Δρ=+0.693), where the 3D model reveals a co-localisation signal that is suppressed to near zero in the 2D analysis. Additionally, Macrophage ↔ Stroma co-localisation (Δρ=+0.511) and CD8^+^ T cell ↔ Other immune co-localisation (Δρ=+0.547) were recovered only under the 3D model, as were conditional exclusions between Other immune ↔ Treg (Δρ=−0.358) and B cell ↔ Treg (Δρ=−0.449). The magnitude of these shared-network deltas in notably larger than in any individual zone, consistent with the shared factors in MSFA aggregating a global spatial signal that accumulates across all zones simultaneously.

In the Very Low tumor density zone, the leading 3D-specific edges were Stroma ↔ Tumor (Δρ=−0.232), Treg ↔ Tumor (Δρ=−0.158), and CD^+^ T cell ↔ Stroma (Δρ=+0.153). The Stroma ↔ Tumor and Treg ↔ Tumor exclusions suggest that in regions of minimal tumor burden, the spatial separation between tumor cells and surrounding stromal and immunosuppressive compartments is a volumetric phenomenon not recoverable from individual tissue sections. The CD8^+^ T cell ↔ Stroma co-localisation recovered in 3D is consistent with cytotoxic T cells trafficking along stromal scaffolding in the peri-tumoral margin, a pattern that would be underestimated when section depth is ignored.

In the Low tumor density zone, the top 3D-specific interactions were CD4+ T cell ↔ Tumor $\left(\Delta \rho =-0.198\right)$, T cell ↔ Tumor (Δρ=+0.142), and CD8^+^ T cell ↔ Stroma (Δρ=+0.133). The sign reversal between CD4^+^ T cell ↔ Tumor and T cell ↔ Tumor within the same zone is notable: the 3D model separates a CD4^+^-specific exclusion from a broader T cell co-localisation signal with tumor, a distinction that collapses entirely in the 2D analysis. This dissociation across T cell subtypes is only resolvable when spatial structure is modeled across the full tissue depth.

In the Intermediate tumor density zone, the dominant 3D-specific edges were T cell ↔ Treg (Δρ=+0.195), Other immune ↔ T cell (Δρ=+0.129), and T cell ↔ Tumor (Δρ=+0.123). All three are co-localisation signals amplified by 3D deconfounding, suggesting that in regions of moderate tumor infiltration the spatial clustering of T cells with immunosuppressive and tumor compartments is organised along the tissue depth axis. The T cell ↔ Treg co-localisation in particular is consistent with active immunosuppressive niche formation that is not apparent from section-level co-occurrence patterns alone.

In the High tumor density zone, the leading 3D-specific edges were T cell ↔ Treg (Δρ=+0.226), T cell ↔ Tumor (Δρ=+0.167), and CD4^+^ T cell ↔ T cell (Δρ=−0.158). The T cell ↔ Treg co-localisation strengthens relative to the Intermediate zone, consistent with progressive immunosuppressive niche consolidation as tumor density increases. Simultaneously, CD4^+^ T cell ↔ T cell exclusion emerges in 3D, indicating that within the broader T cell compartment, CD4^+^ and unlabelled T cells occupy spatially distinct volumetric territories that are indistinguishable in 2D. The B cell ↔ Macrophage co-localisation (Δρ=+0.107) also becomes detectable in 3D, suggesting potential tertiary lymphoid structure organisation in high-density tumor regions.

In the Very High tumor density zone, the three strongest 3D-specific interactions were T cell ↔ Treg (Δρ=+0.272), B cell ↔ Macrophage (Δρ=+0.175), and CD4^+^ T cell ↔ T cell (Δρ=−0.155). The T cell ↔ Treg co-localisation reaches its maximum delta across all zones here, forming a monotonically increasing gradient from Intermediate through Very High tumor density (Δρ=0.195,0.226,0.272 respectively). This gradient is entirely absent in the 2D analysis and represents one of the clearest demonstrations that volumetric spatial modeling recovers biologically coherent, density-dependent immune suppression dynamics that are structurally invisible to section-level analyses. The B cell ↔ Macrophage co-localisation, strengthening from the High to Very High zone, is further consistent with myeloid-lymphoid spatial coupling in the context of dense tumor infiltration.

Taken together, these results demonstrate that a substantial portion of the conditional interaction structure recovered by ISPat-3D is attributable specifically to the Gaussian process deconfounding of the vertical tissue axis and would not be inferred from any analysis operating on individual sections or 2D spatial summaries. The inferences drawn from the delta networks represent relationships that are solely attributable to the 3D component of the pipeline and provide spatial interaction evidence that is categorically inaccessible to conventional 2D multiplexed imaging analyses.

### 3D Breast Cancer Data Description

We also applied ISPat-3D to the three-dimensional imaging mass cytometry (IMC) dataset released by^9^. The dataset comprises a single HER2-positive ductal breast carcinoma (BC) specimen subjected to full volumetric 3D IMC acquisition. Since ISPat-3D requires a true 3D spatial structure with consistent global XY coordinates across registered serial sections and a physical Z axis, this specimen satisfies the structural requirements of the method.

The tissue block was sectioned into 152 consecutive 2μm FFPE slices using an ultramicrotome with a diamond knife, following the protocol described in^9^. Each section was assigned a physical depth coordinate $Z_{\mu \text{m}}=s\times 2$, where s is the section index, yielding a total reconstructed depth of 304μm. IMC acquisition was performed using a panel of 26 metal-conjugated antibodies covering epithelial lineage markers (pan-cytokeratin, CK5, CK7, CK8/18, CK14, CK19, E/P-cadherin, HER2), immune cell markers (CD3, CD8a, CD20, CD45, CD68, CD138), stromal markers (SMA, Vimentin, Collagen I, vWF/CD31), proliferation and apoptosis markers (Ki-67, phospho-H3, pS6, cPARP/cCasp3), and structural markers (Histone H3, Ir193). Images from the overlapping area across all 152 sections were aligned, and single cells were segmented using a 3D watershed algorithm applied to the Ir193 nuclear channel, yielding globally consistent XY coordinates for each segmented cell that are directly comparable across all sections. Mean marker intensities per cell were computed over each single-cell 3D mask and exported as a cell data catalog. Metal isotope spillover between channels was corrected using the compensation matrix described in^9^.

### 3D IMC Breast Cancer Data Analysis

#### ISPat-3D Pipeline

**Figure 7.**
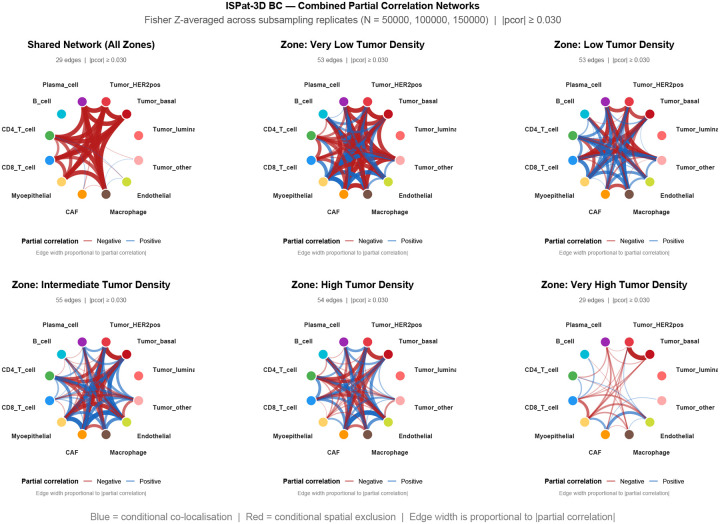
ISPat-3D cell-type interaction networks for the HER2-positive ductal breast carcinoma specimen across tumor density zones. Network diagrams display pairwise partial correlations per network recovered by ISPat-3D. Blue edges indicate positive conditional associations and red edges indicate conditional spatial exclusion. Cell types: Tumor_HER2pos, Tumor_basal, Tumor_luminal, Tumor_other, Endothelial, Macrophage, CAF, Myoepithelial, CD8_T_cell, CD4_T_cell, B_cell, Plasma_cell. Partial correlation values are Fisher Z-averaged across subsampling replicates (N = 25,000; 45,000; 65,000 cells per zone). Only edges with $|\overset{ˆ}{\rho }|\geq 0.030$ are displayed.

To this end, the ISPat-3D framework was applied to jointly model the spatial co-distribution of 12 cell types from this HER2-positive ductal breast carcinoma data: HER2-positive tumor cells, basal tumor cells, luminal tumor cells, other tumor cells, endothelial cells, macrophages, cancer-associated fibroblasts, myoepithelial cells, CD8^+^ T cells, CD4^+^ T cells, B cells, and plasma cells displayed as network diagrams in Figure 7. In this case, the tumor zones were obtained through quantile based partitioning of log normalized pancytokeratin marker intensity. We describe each network in turn, noting where our findings extend beyond the proximity-based analyses reported in^9^ and where the 3D spatial organization of the tissue is necessary to recover the observed associations.

##### Shared network.

The shared network captures zone-invariant conditional associations that reflect the underlying structural biology of the tumor rather than local tumor burden effects. The dominant edge structure in the shared network is a system of strong negative partial correlations, with nearly all associations between major cell classes carrying negative signs. The strongest of these is the Tumor_basal ↔ Tumor_HER2pos exclusion $\left(\overset{ˆ}{\rho }=-1.000\right)$, a conditional anti-correlation that indicates the two dominant tumor subpopulations occupy mutually exclusive spatial territories when all other cell types are held fixed. This is not equivalent to saying the two subtypes never appear in proximity: rather, it reflects that conditioning on the full spatial composition of the tissue, enrichment in one tumor subtype predicts depletion in the other. The structural basis for this is the ductal architecture of the HER2+ carcinoma, in which the HER2-amplified luminal compartment and the CK5+ basal layer form geometrically separated shells around the ductal lumen. A single 2D section through this structure at an oblique angle will intersect both compartments simultaneously, obscuring the conditional exclusion; the 3D reconstruction resolves the spatial shells and makes this exclusion detectable as a network-level phenomenon.

Macrophages are globally negatively associated with all three tumor subtypes in the shared network: Tumor_HER2pos $\left(\overset{ˆ}{\rho }=-0.989\right)$, Tumor_basal $\left(\overset{ˆ}{\rho }=-0.985\right)$, and Plasma_cell $\left(\overset{ˆ}{\rho }=-0.987\right)$. The macrophage-tumor exclusion is consistent with the marginal 3D spatial distributions described in^9^, which showed macrophages concentrated in the stromal compartment rather than within tumor nests, but the conditional nature of the exclusion – that macrophage depletion from tumor regions persists after accounting for the spatial distributions of all other cell types – was not established in that work and cannot be inferred from marginal proximity counts in 2D sections. Similarly, CD4_T_cell is negatively associated with both Tumor_basal $\left(\overset{ˆ}{\rho }=-0.944\right)$ and Tumor_HER2pos $\left(\overset{ˆ}{\rho }=-0.944\right)$, as well as with Macrophage $\left(\overset{ˆ}{\rho }=-0.934\right)$ and Plasma_cell $\left(\overset{ˆ}{\rho }=-0.914\right)$. The CD4_T_cell↔Macrophage negative edge in particular is a finding that is not reported in^9^: it suggests that helper T cells and macrophages are conditionally incompatible spatial neighbors in this tumor, implying they partition the stromal compartment into immunologically distinct territories rather than co-inhabiting the same regions as would be expected in a coordinately immunosuppressive niche. Myoepithelial cells carry strong negative associations with Plasma_cell $\left(\overset{ˆ}{\rho }=-0.912\right)$ and with Macrophage $\left(\overset{ˆ}{\rho }=-0.906\right)$, consistent with the myoepithelial layer forming a physical barrier that separates the intraluminal immune compartment from the stromal immune populations.

##### Very Low tumor density zone.

The Very Low zone network is dominated by CAF interactions, with CAF↔Myoepithelial displaying the strongest positive partial correlation in the entire analysis $\left(\overset{ˆ}{\rho }=0.995\right)$. This edge reflects the structural coupling between cancer-associated fibroblasts and the myoepithelial sleeve of the mammary duct: in the 3D volume, CAFs wrap the outer surface of the myoepithelial layer and form an integrated pericellular scaffold. This association is a fundamentally three-dimensional architectural feature. In a single 2D section, the CAF↔myoepithelial interface is sampled at an arbitrary depth, and its apparent co-localization depends on whether the cutting plane intersects the periductal region. No equivalent quantitative relationship was reported in^9^, whose analysis of basal cell patterning was descriptive and restricted to the morphology of the CK5 layer itself rather than to its fibroblastic microenvironment. The strong positive CAF↔Myoepithelial edge present in the Very Low zone disappears by the Intermediate and High zones, suggesting that CAF↔myoepithelial structural coupling is a feature of regions with low tumor burden where the ductal architecture is more intact, and that this relationship is disrupted as tumor density increases.

CAF is negatively associated with Tumor_other $\left(\overset{ˆ}{\rho }=-0.993\right)$, Tumor_HER2pos $\left(\overset{ˆ}{\rho }=-0.989\right)$, Plasma_cell $\left(\overset{ˆ}{\rho }=-0.972\right)$, and Macrophage $\left(\overset{ˆ}{\rho }=-0.971\right)$ in this zone, while Myoepithelial is positively associated with Tumor_HER2pos $\left(\overset{ˆ}{\rho }=0.975\right)$ and Tumor_other $\left(\overset{ˆ}{\rho }=0.953\right)$. The opposing signs of CAF and Myoepithelial with respect to the tumor compartments indicate a spatial partition in which CAFs and myoepithelial cells form the structural boundary of the duct from opposite sides – myoepithelial cells lining the inner luminal surface and CAFs occupying the outer periductal niche – and that tumor cells are more closely associated with the inner surface than the outer stromal boundary in low-burden regions. The Macrophage↔Tumor_other negative edge $\left(\overset{ˆ}{\rho }=-0.968\right)$ and Plasma_cell↔Tumor_other exclusion $\left(\overset{ˆ}{\rho }=-0.968\right)$ confirm that immune cells are displaced from regions of undifferentiated tumor growth in low-density zones.

##### Low tumor density zone.

The Low zone network shows a major structural reorganization relative to the Very Low zone. CD8_T_cell emerges as the most connected immune cell type: it is negatively associated with Tumor_other $\left(\overset{ˆ}{\rho }=-0.972\right)$, Plasma_cell $\left(\overset{ˆ}{\rho }=-0.897\right)$, and Tumor_HER2pos $\left(\overset{ˆ}{\rho }=-0.870\right)$, while carrying a positive association with Myoepithelial $\left(\overset{ˆ}{\rho }=0.765\right)$. The CD8_T_cell↔Myoepithelial positive conditional association is a novel finding relative to^9^. That paper showed, using 3D rendering, that CD8+ T cells cluster near endothelial structures, but it did not characterize their conditional spatial relationship to myoepithelial cells. Our result suggests that in low tumor burden regions, cytotoxic T cells are preferentially co-localized with the basal compartment of the duct rather than with its HER2-amplified luminal content, which has implications for understanding the entry points of immune cell infiltration into HER2-positive ductal carcinoma.

CD4_T_cell is positively associated with Macrophage $\left(\overset{ˆ}{\rho }=0.811\right)$ in the Low zone, in contrast to the strong negative CD4_T_cell↔Macrophage edge in the shared network. This zone-specific reversal indicates that at low tumor density, CD4 T cells and macrophages are spatially coordinated within the same microenvironmental niches, potentially reflecting collaborative antigen-presenting and T cell priming activity in regions of low tumor burden. As tumor density increases this relationship inverts, suggesting a transition from an immune-coordinating to a spatially partitioned macrophage function. The B_cell↔Endothelial negative association $\left(\overset{ˆ}{\rho }=-0.758\right)$ in the Low zone is absent from both the shared network and the Very Low zone, indicating that in low-burden regions B cells and endothelial cells occupy conditionally exclusive spatial territories – consistent with B cells being organized into lymphoid structures away from vascular beds in this zone – but that this exclusion is not a global feature of the tumor. The CAF↔Tumor_basal positive edge $\left(\overset{ˆ}{\rho }=0.695\right)$ in the Low zone suggests that in low-burden areas CAFs are spatially coupled with basal tumor cells at the ductal boundary rather than excluded from them as in the Very Low zone, reflecting the onset of stromal remodeling that accompanies early tumor expansion.

##### Intermediate tumor density zone.

The Intermediate zone is defined by the emergence of a strong CAF↔Endothelial positive conditional association $\left(\overset{ˆ}{\rho }=0.878\right)$, which was absent from both the Very Low and Low zone networks. This edge reflects the spatial integration of cancer-associated fibroblasts with tumor vasculature at intermediate tumor burden and represents a finding that is not recoverable from 2D analysis. Endothelial channels are tortuous three-dimensional structures whose relationship to the surrounding stromal fibroblast scaffold can only be quantified correctly when the full 3D geometry of the vascular tree is considered. In any given 2D section, the vascular lumen is sampled in cross-section and the periendothelial CAF distribution appears as a simple halo; integrating across the z-axis resolves the coherent co-alignment of CAFs along the vessel axis, which is what the positive conditional association captures. This CAF↔Endothelial coupling persists into the High zone with similar magnitude $\left(\overset{ˆ}{\rho }=0.835\right)$, indicating it is a feature of the tumor microenvironment at elevated tumor burden rather than a transient structural artifact.

The Intermediate zone also exhibits a positive Endothelial↔Tumor_HER2pos association $\left(\overset{ˆ}{\rho }=0.836\right)$ and a positive Endothelial↔Tumor_basal association $\left(\overset{ˆ}{\rho }=0.797\right)$, while CAF carries negative associations with both Tumor_HER2pos $\left(\overset{ˆ}{\rho }=-0.798\right)$ and Tumor_basal $\left(\overset{ˆ}{\rho }=-0.768\right)$. This pattern indicates that in intermediate-burden regions, the vascular compartment is spatially integrated with the tumor compartment while CAFs remain conditionally excluded from it, suggesting that tumor angiogenesis drives endothelial cells into direct spatial coupling with tumor nests while the periductal CAF scaffold is reorganized away from the tumor core. CD4_T_cell carries the strongest negative associations with tumor subtypes in this zone (Tumor_HER2pos: $\overset{ˆ}{\rho }=-0.646$; Tumor_basal: $\overset{ˆ}{\rho }=-0.642$) alongside a positive association with Endothelial $\left(\overset{ˆ}{\rho }=0.639\right)$, suggesting that helper T cells at intermediate tumor density are co-localized with vascular structures rather than with tumor nests, consistent with a perivascular immune accumulation pattern.

##### High tumor density zone.

The High zone network introduces a positive B_cell↔CAF association $\left(\overset{ˆ}{\rho }=0.743\right)$ that is absent from all lower-burden zones and the shared network. This interaction is not reported in^9^ and represents a zone-specific conditional coupling between the B cell and stromal fibroblast compartments that emerges only under elevated tumor burden. In the HER2-positive breast cancer literature, B cells have been associated with tertiary lymphoid structure formation in the stroma, and CAFs have been implicated in organizing the stromal scaffold that supports TLS development. The positive B_cell↔CAF partial correlation observed here is consistent with a model in which TLS-organizing signals between B cells and CAFs are strongest in high tumor burden regions, a spatial hypothesis that could not be generated from the 2D proximity analyses in^9^ because B cell spatial interactions with fibroblasts were not modeled in that study.

CD4_T_cell is negatively associated with Macrophage $\left(\overset{ˆ}{\rho }=-0.610\right)$ in the High zone, reversing the positive CD4↔Macrophage association seen in the Low zone and reinforcing the shared network pattern. The CD8_T_cell↔Endothelial negative association $\left(\overset{ˆ}{\rho }=-0.558\right)$ in the High zone suggests that cytotoxic T cells are conditionally excluded from vascular regions at high tumor density, opposite to the CD4_T_cell↔Endothelial positive association $\left(\overset{ˆ}{\rho }=0.567\right)$ in the same zone. This divergence between CD4 and CD8 T cell relationships to the endothelium at high tumor burden – CD4 cells co-localizing with vessels and CD8 cells excluded from them – is a three-dimensional finding: detecting this differential requires integrating spatial information across the z-axis to correctly estimate distances between T cell populations and the vascular bed, which runs in an orientation that is rarely parallel to any single 2D cutting plane.

##### Very High tumor density zone.

The Very High zone network is structurally sparse compared to all other zones. The strongest edge remains the Tumor_basal↔Tumor_HER2pos negative association $\left(\overset{ˆ}{\rho }=-0.416\right)$, but this effect size is substantially smaller than in lower-burden zones, reflecting the breakdown of coherent spatial organization in regions of maximal tumor burden. The partial correlations in this zone are uniformly weak $\left(|\overset{ˆ}{\rho }|\leq 0.247\right)$, and only 29 edges exceed the threshold in the chord diagram compared to 53–55 in the intermediate zones. This collapse of network structure at the highest tumor density is itself a biologically informative finding: it suggests that the spatial organizational principles governing cell-type co-localization in this tumor – including the CAF↔myoepithelial coupling, the endothelial↔tumor integration, and the immune exclusion patterns – are progressively dismantled as tumor cells come to dominate the microenvironment. This gradient-resolved observation requires the five-zone density stratification that ISPat-3D provides and cannot be detected from any single 2D section, since a given section captures the tumor at one arbitrary depth without reference to the volumetric tumor burden at that location.

The B_cell↔CD8_T_cell positive association $\left(\overset{ˆ}{\rho }=0.110\right)$ and CD4_T_cell↔CD8_T_cell positive association $\left(\overset{ˆ}{\rho }=0.095\right)$ are among the few immune edges surviving in this zone, suggesting that cytotoxic and helper T cell co-localization with B cells represents a residual immune organizational structure that persists even as other spatial relationships degrade under maximal tumor burden.

#### 3D-Specific Volumetric Interactions in BC

**Figure 8.**
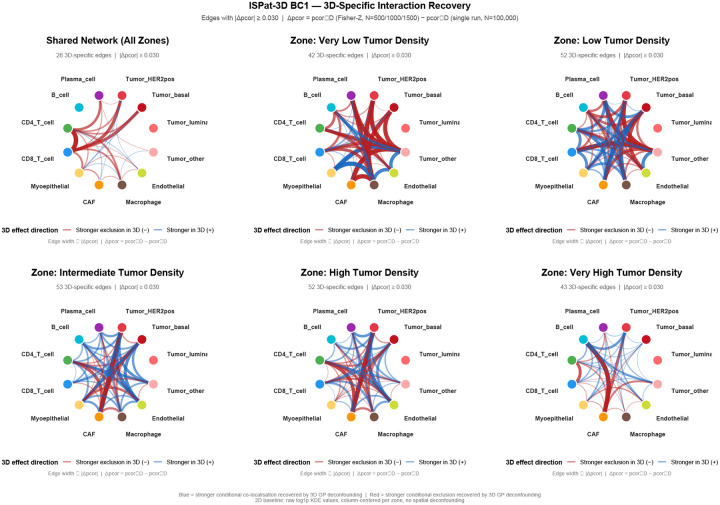
3D-specific interaction networks across tumor density zones in BC1. Each panel displays edges where $\left|\Delta \rho_{ij}\right|\geq 0.030$, defined as $\Delta \rho_{ij}=\rho_{ij}^{3\text{D}}-\rho_{ij}^{2\text{D}}$. Blue edges indicate conditional co-localisations amplified by 3D GP deconfounding; red edges indicate conditional spatial exclusions amplified by 3D deconfounding. Edge width is proportional to $\left|\Delta \rho_{ij}\right|$.

To assess the contribution of three-dimensional spatial deconfounding to network recovery in the BC dataset, we applied the same 2D baseline variant of ISPat-3D used in the CRC1 analysis and applied it to the serial sections of the tissue independently and then combined these networks as before to obtain networks across the tumor density zones. Partial correlation networks from the 3D and 2D analyses were compared at the edge level via delta scores as before. The full set of 3D-specific interaction networks across all five tumor density zones and the shared network are displayed in Figure 8.

In the shared network spanning all zones, the three dominant 3D-specific edges were CD4^+^ T cell ↔ CD88^+^ T cell (Δρ=−1.090), reflecting a strong conditional exclusion between cytotoxic and helper T cell compartments that is completely reversed to apparent co-occurrence in the 2D analysis; CD8^+^ T cell ↔ Tumor_basal_ (Δρ=−0.730), where the 3D model reveals a pronounced spatial segregation between cytotoxic T cells and basal tumor cells that the 2D analysis obscures; and CD8^+^ T cell ↔ Tumor_HER2pos (Δρ=−0.726)_, a similarly strong exclusion between CD8^+^ T cells and the dominant HER2-positive tumor subpopulation that is undetectable from section-level data alone. Additional shared network exclusions recovered exclusively in 3D include CD4^+^ T cell ↔ Plasma cell (Δρ=−0.579), CD4^+^ T cell ↔ Macrophage (Δρ=−0.412), and CD8^+^ T cell ↔ Myoepithelial (Δρ=−0.408). The near-universal sign of shared network 3D-specific edges toward exclusion suggests that the global spatial architecture of this HER2-positive tumor is dominated by volumetric immune segregation from tumor and stromal compartments, a signal that collapses to apparent co-occurrence when section depth is ignored.

In the Very Low tumor density zone, the three leading 3D-specific edges were CAF ↔ Tumor_other_ (Δρ=−1.993), Tumor_HER2pos_ ↔ Tumor_other_ (Δρ=−1.971), and CAF ↔ Macrophage (Δρ=−1.948). These represent complete sign reversals: all three pairs display strong positive partial correlations under the 2D baseline but strong negative partial correlations under the 3D model. The magnitude of these deltas is the largest observed across any zone in either dataset, indicating that in regions of minimal tumor infiltration the spatial organisation of stromal and myeloid compartments relative to tumor cell subpopulations is almost entirely a volumetric phenomenon. Myoepithelial ↔ Tumor_other_ (Δρ=+1.939) and Macrophage ↔ Myoepithelial (Δρ=+1.893) were also recovered as co-localisation signals exclusive to the 3D analysis, suggesting that myoepithelial spatial coupling to both tumor and myeloid populations in the peri-tumoral margin is structured along the tissue depth axis.

In the Low tumor density zone, the top 3D-specific interactions were CD8^+^ T cell ↔ Tumor_other_ (Δρ=−1.782), Tumor_HER2pos_ ↔ Tumor_other_ (Δρ=−1.743), and Plasma cell ↔ Tumor_other_ (Δρ=−1.705). The recurrent involvement of Tumor_other_ across multiple immune and tumor cell exclusions is notable and suggests this subpopulation occupies a spatially distinct volumetric territory that is not resolvable from individual sections. In contrast, CAF ↔ Tumor_basal_ (Δρ=+1.647) and Macrophage ↔ Tumor_basal_ (Δρ=+1.605) emerged as co-localisation signals recovered only under 3D deconfounding, consistent with stromal and myeloid populations tracking basal tumor cell spatial positioning along the depth axis.

In the Intermediate tumor density zone, the three strongest 3D-specific edges were CAF ↔ Tumor HER2pos (Δρ=−1.516), Endothelial ↔ Tumor_HER2pos_ (Δρ=+1.206), and CAF ↔ Tumor_basal_ (Δρ=−1.181). The CAF ↔ Tumor_HER2pos_ and CAF ↔ Tumor_basal_ exclusions reveal that cancer-associated fibroblasts are spatially segregated from both major tumor subpopulations in 3D, despite appearing spatially associated in the 2D analysis. Conversely, Endothelial ↔ Tumor_HER2pos_ co-localisation recovered in 3D is consistent with vascular proximity to HER2-positive tumor cells being organised along the tissue depth axis, likely reflecting angiogenic spatial patterning that is not apparent from section-level co-occurrence. Myoepithelial ↔ Tumor_HER2pos_ (Δρ=+0.878) and CD4^+^ T cell ↔ Endothelial (Δρ=+0.819) co-localisations were also recovered exclusively in 3D, the latter suggesting perivascular CD4^+^ T cell positioning as a volumetric feature of intermediate-density tumor regions.

In the High tumor density zone, the leading 3D-specific interactions were CAF ↔ Tumor_HER2pos_ (Δρ=−1.178), Endothelial ↔ Tumor_HER2pos_ (Δρ=+1.001), and CAF ↔ CD4^+^ T cell (Δρ=−0.906). The CAF ↔ Tumor_HER2pos_ exclusion persists from the Intermediate zone and strengthens, suggesting that stromal segregation from HER2-positive tumor cells is a consistent volumetric feature that intensifies as tumor density increases. The Endothelial ↔ Tumor_HER2pos_ co-localisation similarly persists across Intermediate and High zones (Δρ=+1.206 and +1.001 respectively), forming a zone-consistent 3D-specific vascular proximity signal. CD4^+^ T cell ↔ Macrophage exclusion (Δρ=−0.610) also emerges in this zone, indicating that helper T cell and myeloid compartments are volumetrically separated in high tumor density regions despite apparent 2D co-occurrence.

In the Very High tumor density zone, the three dominant 3D-specific edges were B cell ↔ CAF (Δρ=−0.942), CD4^+^ T cell ↔ CD8^+^ T cell (Δρ=−0.607), and CAF ↔ Tumor_HER2pos_ (Δρ=−0.537). The B cell ↔ CAF exclusion, absent in all other zones, suggests that in the densest tumor regions B cells and cancer-associated fibroblasts occupy non-overlapping volumetric territories, a spatial relationship that is entirely obscured by section-level analysis. The CD4^+^ T cell ↔ CD8^+^ T cell exclusion reappears in this zone after being prominent in the shared network, consistent with T cell subset spatial segregation being a global feature of this tumor that intensifies under maximal tumor burden. B cell ↔ Tumor_HER2pos_ co-localisation (Δρ=+0.484) was also recovered exclusively in 3D, potentially reflecting antibody-mediated antitumor spatial organisation in the context of the highest tumor cell density.

Taken together, the BC1 3D-specific interaction landscape is characterised by two dominant themes that are not recoverable from 2D analysis. The first is a pervasive volumetric segregation of immune effector populations, particularly CD4^+^ and CD8^+^ T cells, from both tumor subpopulations and stromal compartments, a signal that consistently collapses to apparent co-occurrence or neutrality in the 2D baseline. The second is a recurring Endothelial ↔ Tumor_HER2pos_ co-localisation that emerges across Intermediate and High tumor density zones exclusively under 3D deconfounding, consistent with HER2-driven angiogenic spatial coupling that is structured along the tissue depth axis and invisible to section-level analyses. These findings reinforce the conclusion that a substantial portion of biologically interpretable spatial interaction structure in volumetric multiplexed imaging data is accessible only when the vertical tissue axis is explicitly modeled.

## Discussion

We introduced ISPat-3D, a statistical framework for recovering spatially varying cell-type interaction networks from three-dimensional multiplexed tissue images. To our knowledge, this is the first method that explicitly models three-dimensional spatial structure in multiplex imaging data to derive zone-specific partial correlation networks across the tumor microenvironment. Prior spatial interaction methods in computational pathology have operated exclusively in two-dimensional tissue sections, treating each section as an independent observation and discarding the volumetric organization of the tumor. By combining anisotropic Gaussian process regression with multi-study factor analysis, ISPat-3D separates spatially smooth trends from residual co-variation among cell types and then decomposes that residual covariation into a shared network component and zone-specific perturbations. The result is a set of interpretable, conditional interaction maps that vary as a function of local tumor burden, providing a richer characterization of the tumor microenvironment than any single whole-slide summary could offer.

### Biological Relevance for Colorectal Cancer Data

We applied ISPat-3D to the CRC1 specimen from^10^, a poorly differentiated stage IIIB MSI-H colorectal adenocarcinoma for which 3D CyCIF imaging across 25 serial sections produced one of the most spatially detailed single-cell atlases of a human tumor to date.^10^ used a range of spatial statistics to characterize cell-type co-occurrence, nearest-neighbor correlations, and PD1:PDL1 proximity at the invasive margin, and found that T cell suppression at the invasive margin involved multiple cell types, with macrophages playing a central coordinating role in the immunosuppressive architecture of the tumor. Our findings are broadly consistent with this picture and extend it in several respects.

The strongest signal in our shared network, the positive partial correlation between Macrophage and Stroma $\left(\overset{ˆ}{\rho }=0.956\right)$, directly mirrors the observation in^10^ that macrophages in CRC1 are concentrated in stromal compartments and at the invasive margin rather than within tumor nests. The spatial co-localisation of macrophages and stromal cells that our method detects as a conditional association persisting across all five tumor density zones is consistent with the idea that stromal tissue provides the physical niche within which macrophages accumulate and likely exert immunosuppressive function. This association has also been reported in other CRC cohorts, where stromal macrophage infiltration carries adverse prognostic significance.

The strongly negative shared partial correlation between Other immune cells and Treg $\left(\overset{ˆ}{\rho }=-0.953\right)$ and between T cells and Treg $\left(\overset{ˆ}{\rho }=-0.927\right)$ is consistent with the finding from^10^ that T cell suppression at the invasive margin of CRC1 is spatially structured, with regulatory and effector populations occupying partially non-overlapping spatial domains. Conditional on all other cell types, high Treg density co-occurs with lower broad immune infiltration, which is the spatial signature of an immunosuppressive niche. Regulatory T cells are known to erect both physical and functional barriers to effector T cell infiltration through cytokine-mediated suppression, metabolic competition, and stromal remodeling, all of which would manifest as spatial segregation of Tregs from effector immune populations at the tissue level.

In the zone-specific networks, the consistent positive partial correlation between CD4^+^ T cells, CD8^+^ T cells, and Tregs across all five density strata reflects spatially coordinated T cell infiltration that is present even in regions of low tumor burden and persists into areas of maximal tumor density. This co-localisation of helper, cytotoxic, and regulatory T cells is compatible with the organized immune cell clustering at the invasive margins of CRC1. The emergence of B cell co-localisation with CD4^+^ T cells at intermediate and higher tumor densities, which we detect as a positive partial correlation appearing consistently from the Low zone onward, is consistent with the formation of tertiary lymphoid structures or lymphoid aggregates that preferentially arise in regions of sustained immune engagement with the tumor.

A notable feature of our results is the attenuation of effect sizes moving from the shared to the zone-specific networks. Macrophage↔Stroma associations of the magnitude seen in the shared network are not replicated within individual zones at comparable strength, which reflects the fact that the shared component captures the global spatial organization of the tumor microenvironment whereas zone-specific components capture incremental departures from that baseline. This decomposition is methodologically useful: the shared network identifies the architectural 3D skeleton of the TME, and the zone-specific networks reveal how immune and stromal spatial relationships are modulated by local tumor density. Furthermore, the shift in the leading T cell association from CD4^+^↔Treg in the Intermediate and High zones to CD8^+^↔Treg in the Very High zone is consistent with a transition toward cytotoxic T cell suppression at the tumor core.

The B cell↔CD4^+^ T cell spatial co-localisation detected by ISPat-3D from the Low tumor density zone onward has a well-characterized molecular basis. Within tertiary lymphoid structures (TLS), B cells and CD4^+^ follicular helper T cells (Tfh) interact through the CD40↔CD40L receptor-ligand axis, which drives germinal center formation, B cell class switch recombination, and affinity maturation^21–23^. This CD40↔CD40L interaction has been specifically characterized in colorectal cancer, where the strength of this receptor-ligand coupling between germinal center B cells and CXCR5^+^CD4^+^ Tfh cells is significantly higher in early-stage disease and declines in advanced disease, coinciding with impaired TLS maturation^24^. The appearance of this B cell↔CD4^+^ T cell conditional co-localisation at intermediate and high tumor density zones in the CRC1 specimen, detected here for the first time in a 3D spatial network framework, is consistent with TLS precursors forming preferentially in regions of sustained tumor-immune engagement rather than at the lowest tumor density periphery. The second novel finding is the shift in the dominant Treg association from CD4^+^↔Treg at intermediate density to CD8^+^↔Treg at maximal tumor density – has a mechanistic correlate in the IL-2-mediated crosstalk between intratumoral CD8^+^ T cells and Tregs. Research has been conducted demonstrating that activated CD8^+^ T cells produce IL-2 and physically co-localise with Tregs in tumor tissue, promoting Treg accumulation via ICOS upregulation and thereby counteracting their own cytotoxic activity^25^. In colorectal cancer specifically, the ratio of CD8^+^ T cells to intratumoral FoxP3^+^ Tregs is a recognized determinant of antitumor immunity and clinical outcome, with high intratumoral Treg density correlating with suppression of CD8^+^ cytotoxic responses^26^. ISPat-3D recovers this CD8^+^↔Treg spatial proximity as the dominant conditional interaction precisely at maximal tumor density, providing direct spatially resolved evidence at the protein-imaging level for the immunosuppressive niche that characterizes the tumor core.

### Biological Relevance for HER2-Positive Ductal Breast Carcinoma

^9^ characterized this tumor using proximity-based 2D versus 3D distance comparisons, cell phenotype clustering with Phenograph, and qualitative 3D rendering of selected cell populations, but did not model conditional spatial networks between cell types or examine how interaction structure varies across tumor density zones. Our findings extend beyond that descriptive framework in several respects, and several of the key interactions we recover have direct mechanistic correlates in the HER2-positive breast cancer literature.

The dominant feature of the shared network is a comprehensive system of strong negative partial correlations between immune populations and tumor subtypes. All three immune populations with the highest absolute partial correlations in the shared network – Macrophage, CD4^+^ T cells, and CD8^+^ T cells – are negatively associated with both Tumor_HER2pos and Tumor_basal, with effect sizes ranging from $|\overset{ˆ}{\rho }|=0.914$ to $|\overset{ˆ}{\rho }|=0.989$. These large negative values indicate zone-invariant conditional exclusion of immune cells from tumor-occupied regions that persists regardless of local tumor density.^9^ showed that macrophages, CD8^+^ T cells, and B cells were distributed throughout the 3D reconstruction, but their analysis did not account for the spatial partitioning between these populations and the tumor compartment at the conditional network level. The pattern we detect is consistent with an immune-excluded phenotype in this HER2-positive ductal carcinoma, in which physical compartmentalization by the stromal and myoepithelial architecture limits immune cell penetration into tumor-occupied regions. HER2-positive tumors are known to harbor heterogeneous degrees of tumor-infiltrating lymphocytes, and immune exclusion has been mechanistically linked to TGF-β-driven CAF activity and ECM remodeling that restricts T cell migration into tumor epithelium^27,28^.

The strongest novel finding relative to^9^ is the CAF↔Myoepithelial positive partial correlation in the Very Low tumor density zone $\left(\overset{ˆ}{\rho }=0.995\right)$, which is the single largest positive conditional association in the entire analysis. Myoepithelial cells line the inner surface of mammary ducts and are surrounded by a periductal sleeve of cancer-associated fibroblasts that deposit fibronectin, activate TGF-β signaling, and contribute to basement membrane remodeling^29,30^. This periductal CAF↔myoepithelial structural unit has been described as a critical mediator of the transition from ductal carcinoma in situ to invasive ductal carcinoma: tumor-associated myoepithelial cells promote invasive progression by stimulating periductal CAFs to degrade the basement membrane through MMP9 and MMP13 secretion, a process driven by TGF-β/Smads signaling^29,31^. The near-perfect conditional co-localization we detect at low tumor density is consistent with the intact periductal architecture found in low-burden regions where the ductal basal layer has not yet been disrupted. Critically, this association was not quantified in^9^: their analysis described the patchy CK5^+^ basal cell layer morphology in 3D rendering but did not model the conditional spatial coupling between CAFs and the myoepithelial compartment. Furthermore, this structural coupling is a fundamentally three-dimensional observation – in any single 2D section, the CAF↔myoepithelial interface is sampled at an arbitrary depth through the periductal sleeve, and the apparent spatial association depends on whether the cutting plane intersects the periductal region, making recovery of this interaction impossible from 2D data alone.

The strong CAF↔Endothelial positive partial correlation that emerges in the Intermediate $\left(\overset{ˆ}{\rho }=0.878\right)$ and High $\left(\overset{ˆ}{\rho }=0.835\right)$ tumor density zones and persists but weakens in the Very High zone $\left(\overset{ˆ}{\rho }=0.247\right)$ is consistent with an established role for CAFs in supporting tumor angiogenesis through mechanical and paracrine mechanisms. CAFs overexpressing SDF-1/CXCL12 recruit endothelial progenitor cells into breast tumors and stimulate neovascularization through VEGF secretion, and this paracrine cross-talk between fibroblasts and endothelial cells has been demonstrated in 3D vasculogenesis models where breast cancer CAFs drive significantly greater vascular growth than normal fibroblasts through Rho/ROCK-mediated mechanical force transmission^28^. The emergence of this conditional association specifically at intermediate and high tumor burden zones, rather than at low burden, suggests that CAF-mediated angiogenesis is engaged as a tumor density-dependent process in this HER2-positive ductal carcinoma rather than being a constitutive feature of the TME. This zone-resolved observation is only accessible through the volumetric density stratification that ISPat-3D provides: vascular channels are tortuous three-dimensional structures whose co-alignment with the periductal CAF scaffold can only be quantified correctly by integrating spatial information across the z-axis, since any single 2D section intersects vessel lumens at an arbitrary angle that does not capture the longitudinal CAF↔endothelial co-organization.

The B_cell↔CAF positive partial correlation that appears exclusively in the High tumor density zone $\left(\overset{ˆ}{\rho }=0.743\right)$ and is absent from all other zones provides direct spatial protein-level evidence for a structural link between the B cell compartment and the CAF scaffold that has been characterized at the transcriptional level in breast cancer^32,33^. Antigen-presenting CAF subtypes (apCAFs) have been shown to associate closely with tertiary lymphoid structures (TLS) across multiple cancer types, and PDPN^+^ CAFs are capable of organizing the stromal scaffold that supports TLS formation and B cell accumulation in breast cancer^34,35^. In HER2-positive breast cancer specifically, higher TLS density is associated with improved pathological complete response to neoadjuvant therapy, and the presence of B cell-rich immune infiltrates in the stroma has been associated with improved outcomes^36,37^. The zone-specificity of this B_cell↔CAF interaction – appearing only at high tumor burden – is consistent with the hypothesis that TLS formation in this tumor is triggered by elevated antigen load in high-density regions, rather than being a global feature of the microenvironment. This interaction is not recoverable from 2D analysis because B cell spatial relationships to the fibroblast scaffold were not modeled in^9^ and because TLS-organizing spatial structure in 3D runs along depth axes not captured by any single section.

The zone-dependent reversal of the CD4^+^ T cell ↔ Macrophage relationship from positive ($\overset{ˆ}{\rho }=0.811$, Low zone) to negative ($\overset{ˆ}{\rho }=-0.934$, shared network; $\overset{ˆ}{\rho }=-0.610$, High zone) mirrors a well-characterized functional transition in the tumor-associated macrophage compartment across increasing tumor burden. In low-burden regions, macrophages in an immune-priming state engage CD4^+^ T cells through antigen presentation, and the spatial co-localization of M1-polarized macrophages with helper T cells at sites of immune priming is consistent with the positive partial correlation we observe in the Low zone^38^. As tumor density increases, tumor-associated macrophages transition toward an M2-polarized immunosuppressive phenotype that actively excludes CD4^+^ T cells from their spatial niche through TGF-β, IL-10, and checkpoint ligand signaling^39^. The reversal of the CD4^+^ T cell ↔ Macrophage interaction sign from positive to negative across the zone spectrum is a gradient-resolved observation that requires volumetric density zonation to detect: in any single 2D section, macrophage functional state varies along the z-axis with tumor depth, and integrating information from a flat image conflates immune-priming and immunosuppressive macrophage spatial contexts into a single marginal density estimate that cannot resolve this functional transition.

The divergence between CD4^+^ T cells and CD8^+^ T cells in their relationship to the endothelium in the High zone – CD4^+^ T cells conditionally co-localizing with endothelial cells $\left(\overset{ˆ}{\rho }=0.567\right)$ while CD8^+^ T cells are conditionally excluded from the endothelial compartment $\left(\overset{ˆ}{\rho }=-0.558\right)$ – is consistent with an immune exclusion pattern in which cytotoxic T cells are blocked from engaging the perivascular niche while helper T cells accumulate near vascular structures. Perivascular tumor-associated macrophages occupy niches immediately adjacent to blood vessels and have been shown to actively suppress CD8^+^ T cell infiltration while permitting CD4^+^ T cell and regulatory T cell accumulation in the perivascular space^40,41^. This spatial segregation of CD4^+^ and CD8^+^ T cells relative to the vasculature is a three-dimensional organizational feature of the tumor: endothelial channels run along axes that are not parallel to any standard 2D section plane, and the differential perivascular distribution of T cell subsets can only be characterized by measuring distances from cells to the full 3D vascular network rather than to 2D cross-sections of vessel lumens.

Finally, the progressive collapse of network complexity from the Intermediate zone (55 edges above threshold) to the Very High zone (29 edges above threshold), accompanied by a reduction in partial correlation magnitudes across all edges in the Very High zone, is itself a biologically informative finding. In regions of maximal tumor burden, the spatial organizational principles that govern cell-type co-localization in this tumor – the CAF↔myoepithelial structural coupling, the CAF↔endothelial angiogenic co-organization, the immune exclusion patterns, and the zone-specific B cell↔CAF interaction – are collectively attenuated, suggesting that extreme tumor density in HER2-positive ductal carcinoma disrupts the tissue architecture that normally enforces these conditional spatial relationships. This gradient-resolved observation is accessible only through the volumetric tumor density zonation that ISPat-3D provides, and cannot be detected from any single 2D section since a flat image captures the tumor at one arbitrary depth without reference to the volumetric tumor burden at that location.

### Methodological Contributions and Novelty

Existing spatial analysis tools for multiplexed imaging, including co-occurrence statistics, spatial cross-correlation functions, and cell neighborhood methods, operate on discrete cell-level event data in 2D and do not account for spatial autocorrelation when estimating cell-type associations. ISPat-3D addresses both limitations. The GP regression stage explicitly removes spatially smooth trends from each cell-type intensity surface, so that the covariation passed to MSFA reflects residual spatial association rather than a confound introduced by the global arrangement of tissue compartments. The MSFA component then separates associations that are common to all zones from those that are zone-specific, enabling a decomposition that is unavailable from any single-zone or whole-slide analysis. The use of an anisotropic Matérn kernel with separate lateral and axial lengthscale parameters is essential for 3D imaging data, where the imaging resolution and physical scale differ substantially between the xy plane and the z axis across serial sections. We have applied this method to two 3D multiplexed cancer datasets to understand spatially resolved ligand receptor interactions varying over tumor burden. This method can also be applied to any 3D spatial dataset with multiple variables (e.g., cell types, genes, voxel intensity values, fluorescence intensity, radiomic features) and arbitrary annotations (e.g., tumor specific: invasive front, tumor core, tumor margin, peritumoral region, leading edge; vascular and necrotic specific: necrotic core, perinecrotic zone, hypoxia-induced region, angiogenic hotspot, microvascular proliferation) to understand the volumetric architecture of these variables. This makes ISPat-3D the first method of its kind to present itself as a generalizable framework across different domains. We have tested this method in the case where the number of variables is less than the sample size (p<n), but this can be directly applied to high dimensional settings where p>n and we expect similar performance.

### Limitations

The primary practical limitation of ISPat-3D as applied here is computational. The CRC1 3D dataset contains on the order of 2 × 10^8^ segmented cells across the 25 imaged sections, a scale entirely beyond the reach of standard Gaussian process inference, which has $𝒪\left(N^{3}\right)$ time and $𝒪\left(N^{2}\right)$ memory complexity in the number of observations N. A similar issue in the BC 3D dataset was observed as well. We addressed this through spatially stratified subsampling, drawing 50000, 100000, and 150000 or some other number of cells per zone and aggregating results via Fisher Z-transform averaging of partial correlation matrices across replicates. While this approach is statistically principled, it introduces a dependence on the spatial coverage provided by the subsample and cannot exploit the full statistical information in the complete cell population. Sparse GP approximations, inducing point methods, or scalable variational GP inference could in principle reduce this burden and allow ISPat-3D to operate on larger cell populations without subsampling; this remains an open problem for future research. The shared network exhibited slightly higher variability across subsampling replicates than the zone-specific networks, which is expected because shared factor estimation draws on all zones simultaneously and inherits variance from each. A second limitation is that the current pipeline treats each zone independently in the GP regression stage before pooling through MSFA. A fully joint model that smooths across zones would in principle improve estimation for zones with sparse cell coverage, but at substantially increased model complexity and computational cost. Finally, the analysis presented here is based on the CRC1 and BC-HER2 diseased independent specimens which shows that this method is generalizable. The interaction maps recovered from the 3D analysis substantially differ from the baseline 2D analysis, which strengthens our analysis and provides volumetric understanding of cell colocalization and functions. While the BC data was less characterized for exploration, CRC1 is a well-characterized, high-complexity tumor with MSI-H status and a multi-front invasive margin, making it an informative test case, but the generalizability of these network structures requires validation across additional 3D specimens for each diseased tumor under consideration. Extension to the remaining 16 CRC whole-slide specimens available in the^10^ cohort, as well as to other tumor types for which 3D multiplexed imaging is now feasible, represents immediate next steps for ISPat-3D. Lastly, all the interactions ↔ considered here are from an undirected graph, i.e., they are bidirectional in nature; future research also includes the understanding of directionality and subsequent causal nature of these cell type functions in the 3D spectrum.

## Methods

### ISPat-3D: Informed Spatially Aware Patterns in Three Dimensions

#### Overview of the Modeling Framework

ISPat-3D is a hierarchical Bayesian framework designed to characterize how cell-cell interaction patterns vary across the tumor intensity gradient within a tissue image. The model operates in three sequential stages within each image: (i) zone-specific Gaussian process (𝒢𝒫) regression to recover latent spatial expression surfaces from raw marker intensities or cell type specific kernel density estimates, (ii) multi-study factor analysis (MSFA) across the five tumor zones to decompose covariance into shared and zone-specific components, and (iii) network construction from the resulting precision matrices to represent conditional co-localization structure among cell types. These three stages together constitute the within-image inference.

#### Notation and Data Structure

Let G denote the number of cell type-expressions, and let N be the total number of cells in image ℐ. The data for a single image consist of a G×N cell type intensity matrix and an N×3 coordinate matrix S, where the three columns give the spatial coordinates $s_{j}={\left(s_{x},s_{y},s_{z}\right)}_{j}$ and zone specific annotation that gives the tumor zone label q∈{1,…,5}. For zone q, let ${𝒫}_{q}\subset \{1,\ldots ,N\}$ denote the index set of cells assigned to ${ℐ}_{q}$, so that $N_{q}=\left|{𝒫}_{q}\right|$.

#### Joint Distribution of Cell-Type Intensities Across Spatial Regions

We characterize the spatial co-localization structure among G cell type intensities by modeling their joint intensity distribution within each of the Q tumor intensity-defined regions. For a given tissue image ℐ, the cell intensity profile at spatial location $s_{j}\in {ℐ}_{q}$ is the G-dimensional vector $f_{q}\left(s_{j}\right)={\left\{f_{q}^{1}\left(s_{j}\right),f_{q}^{2}\left(s_{j}\right),\ldots ,f_{q}^{G}\left(s_{j}\right)\right\}}^{⊤}$, where $f_{q}^{g}\left(s_{j}\right)$ denotes the latent intensity of cell g at location $s_{j}$ within region ${ℐ}_{q}$, corresponding to the g-th row of Y restricted to cells ${𝒫}_{q}$. The joint distribution of these intensities is modeled as multivariate Gaussian:

(2) $$f_{q}\left(s_{j}\right)\sim ℳ𝒩\left(\mu_{q},{𝚺}_{q}\right),\;j\in {𝒫}_{q},\;q=1,\ldots ,Q,$$

where $\mu_{q}\in R^{G}$ is the region-specific mean intensity vector and ${𝚺}_{q}\in R^{G\times G}$ is the region-specific covariance matrix encoding pairwise co-localization relationships among all G cells within ${ℐ}_{q}$. The total number of cells satisfies $N=\sum_{q=1}^{Q}N_{q}$ where $N_{q}=\left|{𝒫}_{q}\right|$.

The covariance matrix ${𝚺}_{q}$ is the primary inferential target at this stage. Its $\left(g,g^{\prime }\right)$ entry ${𝚺}_{q}^{\left(gg^{\prime }\right)}$ quantifies the degree to which the intensities of cells g and $g^{\prime }$ co-vary across spatial locations within region ${ℐ}_{q}$, capturing spatial co-localization when positive and spatial exclusion when negative. The corresponding precision matrix ${𝛀}_{q}={𝚺}_{q}^{-1}\in R^{G\times G}$ encodes conditional independence structure: a zero entry ${𝛀}_{q}^{\left(gg^{\prime }\right)}=0$ implies that cells g and $g^{\prime }$ are conditionally independent given the intensities of all remaining cells within ${ℐ}_{q}$, i.e., $f_{q}^{g}⫫f_{q}^{g^{\prime }}|f_{q}^{\setminus \left(g,g^{\prime }\right)}$. Conversely, a non-zero entry ${𝛀}_{q}^{\left(gg^{\prime }\right)}\neq 0$ indicates a direct conditional association between cells g and $g^{\prime }$ that is not explained by their mutual relationships with other cells, and corresponds to an edge $\left(g,g^{\prime }\right)$ in the undirected interaction graph ${𝒢}_{q}=\left(V,E_{q}\right)$ with vertex set V={1,…,G}.

This precision-based graph representation is preferable to a marginal correlation network for characterizing cell co-localization in CyCIF and IMC datasets because it filters out spurious associations driven by shared spatial variation, retaining only direct pairwise dependencies. The Gaussian Markov random field (GMRF) structure implied by ${𝛀}_{q}$ thereby provides a parsimonious and interpretable summary of the local cellular interaction architecture within each tumor zone. We do not estimate ${𝚺}_{q}$ directly from the $N_{q}\times G$ submatrix of Y restricted to ${𝒫}_{q}$, as direct estimation would be unreliable when spatial autocorrelation inflates the effective sample size and when $N_{q}$ is moderate relative to G. Instead, ${𝚺}_{q}$ is recovered implicitly through the zone-specific 𝒢𝒫 denoising in Stage 1 and the MSFA decomposition in Stage 2, which jointly account for spatial dependence and borrow strength across zones via the shared loading structure 𝚽.

#### Stage 1: Zone-Specific Anisotropic Gaussian Process Regression

Within each zone q and for each cell g=1,…,G, we model the observed intensity $f_{q}^{g}\left(s_{j}\right)$ at cell $j\in {𝒫}_{q}$ as a sum of three components:

(3) $$f_{q}^{g}\left(s_{j}\right)=\beta_{g}+\xi_{g}^{q}\left(s_{j}\right)+\zeta_{g}^{q}\left(s_{j}\right)+\varepsilon_{j},\;j\in {𝒫}_{q},$$

where $\beta_{g}\in R$ is a global intercept for cell $g,\xi_{g}^{q}(s)\sim 𝒢𝒫\left(0,{𝒦}_{q}(\cdot ,\cdot )\right)$ is a spatially correlated nuisance field encoding local intensity variation within zone $q,\zeta_{g}^{q}\left(s_{j}\right)$ is a spatially unstructured signal drawn from a cross-cell covariance that captures cell-cell interaction patterns and is the target of Stage 2 MSFA, and $\varepsilon_{j}\sim 𝒩\left(0,\sigma_{\varepsilon }^{2}\right)$ is an independent nugget term encoding measurement noise. The GP stage estimates and removes $\xi_{g}^{q}$; the residual $f_{q}^{g}-{\overset{ˆ}{\beta }}_{g}-{\overset{ˆ}{\xi }}_{g}^{q}$ is then passed to MSFA.

##### Kernel specification.

ISPat-3D supports two anisotropic covariance kernels that treat the (x,y) tissue plane and the z-axis with separate lengthscales $\ell_{S}$ and $\ell_{Z}$ respectively. For cells $s_{j}$ and $s_{j^{\prime }}$ in zone q, the anisotropic distance is

(4) $$r\left(s_{j},s_{j^{\prime }}\right)=\sqrt{{\left(\frac{s_{jx}-s_{j^{\prime }x}}{\ell_{S}}\right)}^{2}+{\left(\frac{s_{jy}-s_{j^{\prime }y}}{\ell_{S}}\right)}^{2}+{\left(\frac{s_{jz}-s_{j^{\prime }z}}{\ell_{Z}}\right)}^{2}}.$$

The Matérn-3/2 kernel is then

(5) $${𝒦}_{\text{Mat}}\left(s_{j},s_{j^{\prime }}\right)=\sigma_{S}^{2}\left(1+\sqrt{3}r\right)\text{exp}\left(-\sqrt{3}r\right),$$

and the squared exponential (RBF) kernel is

(6) $${𝒦}_{\text{RBF}}\left(s_{j},s_{j^{\prime }}\right)=\sigma_{S}^{2}\text{exp}\left(-\frac{r^{2}}{2}\right).$$

The Matérn-3/2 is preferred in practice because it imposes once-differentiable sample paths, which is a more realistic smoothness assumption for cell-level spatial processes than the infinitely differentiable RBF. Positive definiteness of the kernel matrix is verified at runtime and enforced via nearest positive definite projection when necessary. This particular choice of lengthscale parameterization enables us to capture the volumetric notion of the 3D data. Given that these 3D images are serial sections of an actual volumetric tumor, the anisotropic kernel takes the geometric axial direction of variation into account in each spatial regression.

##### Hyperparameter estimation.

The hyperparameter vector $\theta_{g}^{q}=\left(\ell_{S},\ell_{Z},\sigma_{S},\sigma_{\varepsilon },\beta_{g}\right)$ is estimated jointly by maximizing the GP log marginal likelihood, obtained by integrating out the latent field $\xi_{g}^{q}$ analytically. Letting $V_{q}=\sigma_{S}^{2}K_{q}+\sigma_{\varepsilon }^{2}I_{N_{q}}$, the log marginal likelihood is

(7) $$\text{log}\;p\left(f_{q}^{g}|\theta_{g}^{q}\right)=-\frac{1}{2}{\left(f_{q}^{g}-\beta_{g}1\right)}^{⊤}V_{q}^{-1}\left(f_{q}^{g}-\beta_{g}1\right)-\frac{1}{2}\text{log}\left|V_{q}\right|-\frac{N_{q}}{2}\text{log}\;2\pi .$$

This type-II maximum likelihood objective is optimized via L-BFGS-B^42,43^ with parameters reparameterized on the log scale to enforce positivity. Lengthscale bounds are anchored to the empirical spatial range of zone q: the lower bound is set to one tenth of the median pairwise distance and the upper bound to ten times the median, separately for the (x,y) plane and the z-axis. This data-adaptive bounding avoids degenerate solutions at near-zero lengthscales, which correspond to exact interpolation, or at lengthscales exceeding the domain extent, which are unidentifiable from the data^44^. The marginal likelihood approach subsumes the need for cross-validation at the per-zone, per-cell level and is standard practice in spatial transcriptomics^13^.

##### Latent field estimation.

The observed intensity for cell g in zone q decomposes as

(8) $$f_{q}^{g}={\overset{ˆ}{\beta }}_{g}1_{N_{q}}+{\overset{ˆ}{B}}_{g}^{q}+{\overset{ˆ}{z}}_{g}^{q},$$

where ${\overset{ˆ}{B}}_{g}^{q}$ is the estimated spatial nuisance and ${\overset{ˆ}{z}}_{g}^{q}$ is the spatially adjusted residual that carries the cross-cell network structure subsequently analysed by MSFA. Given the optimized hyperparameters ${\overset{ˆ}{\theta }}_{g}^{q}$, the posterior mean of the spatial nuisance field is recovered via the standard conditional Gaussian identity^44^:

(9) $${\overset{ˆ}{B}}_{g}^{q}={\overset{ˆ}{\sigma }}_{S}^{2}K_{q}V_{q}^{-1}\left(f_{q}^{g}-{\overset{ˆ}{\beta }}_{g}1_{N_{q}}\right),$$

where $V_{q}={\overset{ˆ}{\sigma }}_{S}^{2}K_{q}+{\overset{ˆ}{\sigma }}_{\varepsilon }^{2}I_{N_{q}}$. The MSFA input is then the spatially adjusted residual

(10) $${\overset{ˆ}{z}}_{g}^{q}=\left(f_{q}^{g}-{\overset{ˆ}{\beta }}_{g}1_{N_{q}}\right)-{\overset{ˆ}{B}}_{g}^{q}={\overset{ˆ}{\sigma }}_{\varepsilon }^{2}V_{q}^{-1}\left(f_{q}^{g}-{\overset{ˆ}{\beta }}_{g}1_{N_{q}}\right),$$

which retains only the non-spatial structured variation across cells. Stacking across G cells yields the spatially adjusted matrix ${\overset{ˆ}{Z}}^{q}\in R^{N_{q}\times G}$, which is passed to MSFA as the observed input for zone q. The solve is implemented via Cholesky decomposition for numerical stability. Estimation across the G cells within each zone is parallelized across available cores.

#### Stage 2: Multi-Study Factor Analysis Across Tumor Zones

After Stage 1, each image yields five zone-specific latent matrices ${\left\{Z^{q}\right\}}_{q=1}^{5}$, each of dimension $N_{q}\times G$. These matrices are treated as the data inputs to a multi-study factor analytic (MSFA) model that decomposes the cross-cell-type covariance into a component shared across all five CK zones and five zone-specific residual components. The biological motivation is direct: cell-cell interaction patterns that persist regardless of local tumor density reflect constitutive structural features of the tumor microenvironment, whereas zone-specific patterns capture how interactions rewire along the tumor gradient.

The MSFA model for zone q at cell $j\in {𝒫}_{q}$ is

(11) $$f_{q}\left(s_{j}\right)=𝚽F_{qj}+{𝚲}_{q}D_{qj}+\epsilon_{qj},$$

where $𝚽\in R^{G\times K}$ is the shared loading matrix with K=⌊2logG⌋ latent factors common to all zones, $F_{qj}\sim {𝒩}_{K}\left(0,I_{K}\right)$ are shared factor scores, ${𝚲}_{q}\in R^{G\times K_{s}}$ with $K_{s}=\lfloor 2\;\text{log}\;G\rfloor$ is the zone-specific loading matrix, $D_{qj}\sim {𝒩}_{K_{s}}\left(0,I_{K_{s}}\right)$ are zone-specific scores, and $\epsilon_{qj}\sim 𝒩\left(0,{𝚿}_{q}\right)$ with ${𝚿}_{q}=\text{diag}\left(\psi_{q1}^{2},\ldots ,\psi_{qG}^{2}\right)$. The implied zone-level covariance matrix is

(12) $${𝚺}_{q}=𝚽{𝚽}^{⊤}+{𝚲}_{q}{𝚲}_{q}^{⊤}+{𝚿}_{q}.$$

Inference is performed either via MCMC, coordinate ascent variational inference (CAVI), or stochastic variational inference (SVI), all of which yield posterior means $\overset{ˆ}{𝚽}$ and ${\left\{{\overset{ˆ}{𝚲}}_{q}\right\}}_{q=1}^{5}$ as the primary inferential targets. For our inference task we employ MSFA using CAVI.

#### Stage 3: Network Construction

The shared interaction network and five zone-specific interaction networks are constructed from the MSFA loading matrices as follows. The shared network captures cell-type co-localization patterns that are consistent across all CK zones:

(13) $${\hat{W}}_{\text{shared}}=\overset{ˆ}{𝚽}{\overset{ˆ}{𝚽}}^{⊤}\in R^{G\times G}.$$

The zone-specific network for zone q augments the shared component with the zone-specific deviation:

(14) $${\hat{W}}_{q}={\overset{ˆ}{𝚲}}_{q}{\overset{ˆ}{𝚲}}_{q}^{⊤}+\overset{ˆ}{𝚽}{\overset{ˆ}{𝚽}}^{⊤}+{𝚿}_{q}\in R^{G\times G},\;q=1,\ldots ,5.$$

In both cases the $\left(g,g^{\prime }\right)$ entry of $\hat{W}$ encodes the strength of the functional co-localization relationship between cell types g and $g^{\prime }$, with non-zero entries corresponding to edges in the undirected graph 𝒢=(V,E) where V={1,…,G}. The conditional independence interpretation follows directly from the Gaussian Markov random field framework: $\left(g,g^{\prime }\right)\notin E⟺f_{q}^{g}╨f_{q}^{g^{\prime }}|f_{q}^{\setminus \left(g,g^{\prime }\right)}$, which under the multivariate Gaussian model is equivalent to a zero in the precision matrix ${𝛀}_{q}={𝚺}_{q}^{-1}$.

#### Reference-Based Covariance Construction from Adjacency Structure

A key modeling decision in ISPat-3D concerns the representation of cell-cell or cell marker-cell marker interaction structure. Adjacency matrices, while natural summaries of graph topology, cannot be directly used as covariance matrices within a Gaussian probabilistic framework because they are not guaranteed to be positive semidefinite and do not encode the magnitude of co-expression relationships beyond binary connectivity. We therefore adopt a covariance-based representation throughout, constructing valid positive semidefinite matrices from the MSFA as described in the Stage 2 section. However, when prior biological knowledge about cell-cell or cell marker-cell marker adjacency is available, for instance from receptor-ligand databases or spatial proximity graphs derived from the tissue, it is natural to ask whether such adjacency information can be incorporated to regularize or inform the estimated covariance patterns. We describe a principled transformation that achieves this.

Let $A\in R^{G\times G}$ denote a symmetric adjacency matrix encoding known or hypothesized interactions among G cell types, with $A_{gg^{\prime }}\in \{0,1\}$ or more generally $A_{gg^{\prime }}\geq 0$. To convert A into a valid reference covariance matrix ${𝚺}^{*}\in R^{G\times G}$, we exploit the additive decomposition of Gaussian process covariance functions. A function h(x) defined over a product domain can be written in an additive form as $h(x)=c+\sum_{i}h_{i}\left(x_{i}\right)$, where each component $h_{i}$ is a univariate function of a single input dimension^45^. Under a Gaussian process prior, additive functions of this form correspond to additive covariance kernels, and the sum of valid covariance kernels is itself a valid covariance kernel^44,46^. This additive 𝒢𝒫 structure justifies constructing a reference covariance by treating the adjacency matrix as a linear combination of positive semidefinite rank-one components, each corresponding to a pairwise interaction term.

Formally, the reference covariance matrix is obtained by projecting A onto the cone of symmetric positive semidefinite matrices. One natural choice is to set ${𝚺}^{*}=A+\delta I_{G}$ for a small regularization constant δ>0 chosen to ensure positive definiteness, provided the resulting matrix has non-negative eigenvalues after regularization. A more principled alternative, particularly when A may have negative eigenvalues due to its graph Laplacian structure, is to take the nearest positive definite matrix to A in the Frobenius norm sense^47^, or to construct ${𝚺}^{*}$ directly from the spectral decomposition of A by retaining only positive eigenvalues.

Given a valid reference covariance ${𝚺}^{*}$, the reference-based zone-specific interaction pattern for region ${ℐ}_{q}$ is defined as

(15) $${\tilde{W}}_{q}={\left[{𝚺}^{*}\right]}^{1/2}{𝚲}_{q}{𝚲}_{q}^{⊤}{\left[{𝚺}^{*}\right]}^{1/2},$$

where ${\left[{𝚺}^{*}\right]}^{1/2}$ denotes the symmetric matrix square root of ${𝚺}^{*}$, obtained via eigendecomposition as ${\left[{𝚺}^{*}\right]}^{1/2}=U{𝚫}^{1/2}U^{⊤}$ with ${𝚺}^{*}=U𝚫U^{⊤}$. The construction in Equation (15) has a direct interpretation: it rescales the zone-specific loading outer product ${𝚲}_{q}{𝚲}_{q}^{⊤}$ by the reference covariance structure, so that pairs of cell types with strong prior adjacency contribute more heavily to the zone-specific pattern than pairs with weak or absent adjacency. This acts as a soft structural prior on the network, preserving the data-driven zone-specific variation encoded in ${𝚲}_{q}$ while anchoring the interaction magnitude to a biologically informed reference scale.

Note that ${\tilde{W}}_{q}$ is positive semidefinite by construction, since for any vector $v,v^{⊤}{\tilde{W}}_{q}v={\left\|{𝚲}_{q}^{⊤}{\left[{𝚺}^{*}\right]}^{1/2}v\right\|}^{2}\geq 0$. This ensures that ${\tilde{W}}_{q}$ remains a valid covariance matrix that can be passed to downstream precision matrix estimation or graph inference procedures. The analogous reference-based shared network is

(16) $${\tilde{W}}_{\text{shared}}={\left[{𝚺}^{*}\right]}^{1/2}𝚽{𝚽}^{⊤}{\left[{𝚺}^{*}\right]}^{1/2},$$

which regularizes the shared interaction structure by the same reference covariance. In settings where no prior adjacency information is available, one sets ${𝚺}^{*}=I_{G}$, recovering the unregularized estimators ${\hat{W}}_{q}={𝚲}_{q}{𝚲}_{q}^{⊤}+𝚽{𝚽}^{⊤}+{𝚿}_{q}$ and ${\hat{W}}_{\text{shared}}=𝚽{𝚽}^{⊤}$ already described above.

The output of ISPat-3D for a single image is therefore one shared network and five zone-specific networks, together providing a complete characterization of how cellular interaction structure varies from low to high tumor density within the tissue. These interaction patterns are obtained as conditional independence structures that vary over tumor trajectory keeping the volumetric feature of the 3D data in account. Given the formulation of ISPat-3D, we can now uncover the ligand receptor interaction graphs which are not captured via any method built for 2D image analysis.

## Data Availability

The CRC1 CyCIF dataset is publicly available from the Lin et al. (2023) data release. The 3D IMC breast carcinoma dataset is available from Zenodo (https://doi.org/10.5281/zenodo.4752030).

## Code availability

All analysis code is available at https://github.com/sagnikbhadury/ISPAT-3D.

## Appendix A: CRC Data Processing

### Acquisition and column standardization.

Per-section cell feature tables were downloaded for all 25 CRC1 serial sections. Each feature table was standardized to a common schema. Global tissue-level coordinates $\left(X_{s},Y_{s}\right)$ from the registered whole-slide images were used directly as the canonical XY position for each cell, ensuring spatial consistency across sections. A panel of 23 markers was retained: epithelial markers (Keratin, E-cadherin, CDX2), immune lineage markers (CD45, CD3, CD4, CD8a, FOXP3, CD20, CD68, CD163, CD45RO), proliferation markers (Ki67, PCNA), stromal markers (α-SMA, Vimentin, Desmin, Collagen, CD31), checkpoint markers (PDL1, PD1), and structural markers (NaKATPase, LaminABC). Marker intensity values were winsorized at the 99.9th percentile to mitigate the influence of imaging outliers. Cells with missing XY coordinates or any missing marker intensity after winsorization were excluded, as were sections with fewer than 1000 retained cells.

### Cell type classification.

Cell types were assigned using a hierarchical threshold-based gating strategy applied independently to each section, following the classification framework of^10^. Nine cell types were defined: tumor cells (Keratin > 7000, E-cadherin > 3500, or CDX2 > 15000), regulatory T cells (FOXP3 > 200 and CD4 > 1900), CD^+^ cytotoxic T cells (CD8a > 2500), CD4^+^ helper T cells (CD4 > 1900), general T cells (CD3 > 5800), B cells (CD20 > 3300), macrophages (CD163 > 760 or CD68 > 430), other immune cells (CD45 > 2000), and stromal cells (α-SMA > 3300, Vimentin > 560, or Collagen > 7700). Labels were assigned in the listed order of precedence, so that more specific immune phenotypes supersede broader lineage assignments. Cells satisfying no criterion were labeled unclassified and excluded from all downstream analyses.

### Spatial zone annotation via tumor cell density partitioning.

Tumor cell spatial density serves as a biologically meaningful stratification axis for partitioning each tissue section into spatially coherent microenvironmental compartments. Tumor cell density varies systematically across a tissue section, encoding the spatial gradient from immune-rich, tumor-sparse stromal and peri-tumoral regions to dense tumor epithelial cores. This gradient is particularly informative in the context of CRC, where the spatial organization of immune and tumor cell compartments differs substantially across the invasive margin morphologies documented in CRC1. We therefore estimate a spatially smoothed tumor cell density surface via two-dimensional kernel density estimation (KDE) and use it as the stratification axis to partition each section into biologically interpretable spatial domains prior to downstream inference.

For each section, the XY coordinates of all classified tumor cells were extracted and a Gaussian KDE surface was fitted on a 200 × 200 grid spanning the full XY extent of the section. The kernel bandwidth was selected separately along each spatial axis by leave-one-out cross-validation (LOOCV). Specifically, for a univariate coordinate vector $x=\left(x_{1},\ldots ,x_{n}\right)$, the LOOCV objective is:

(17)
$$\hat{h}=\text{arg}\;\underset{h}{\text{min}}\left\{-\sum_{i=1}^{n}\text{log}\left[\frac{1}{n-1}\sum_{j\neq i}\frac{1}{h}\phi \left(\frac{x_{i}-x_{j}}{h}\right)\right]\right\},$$

where ϕ(⋅) denotes the standard Gaussian density. The search interval for h was set to $\left[0.05h_{\text{Scott}},5h_{\text{Scott}}\right]$, where $h_{\text{Scott}}=1.06\hat{\sigma }n^{-1/5}$ is the Scott bandwidth^48^, and optimization was carried out using Brent’s method^49^. When the number of tumor cells in a section exceeded 5000, a random subsample of 5000 cells was used for bandwidth estimation while the full tumor cell set was retained for KDE fitting. Sections with fewer than 50 tumor cells were assigned uniformly to zone 1.

The fitted KDE surface was bilinearly interpolated at the XY location of every cell j in the section, regardless of cell type, yielding a continuous local tumor density score ${\hat{\kappa }}_{j}$. For each tissue section ℐ, let ${\left\{{\hat{\kappa }}_{j}\right\}}_{j=1}^{N}$ denote these scores across all N cells at spatial locations $\left\{s_{j}\right\}$. We partition ℐ into five non-overlapping zones ${\left\{{ℐ}_{q}\right\}}_{q=1}^{5}$ by thresholding on the empirical quantiles of $\left\{{\hat{\kappa }}_{j}\right\}$:

(18)
$${ℐ}_{q}=\left\{s_{j}\in ℐ:F_{N}^{-1}\left(\frac{q-1}{5}\right)\leq {\hat{\kappa }}_{j}<F_{N}^{-1}\left(\frac{q}{5}\right)\right\},\;q=1,\ldots ,5,$$

where $F_{N}^{-1}$ is the empirical quantile function of $\left\{{\hat{\kappa }}_{j}\right\}$ across all N cells, and the closed upper bound in the final stratum ensures no cell is excluded at the maximum density value. The five zones are labeled Very Low (q=1,0−20th percentile), Low (q=2,20−40th percentile), Intermediate (q=3,40−60th percentile), High (q=4,60−80th percentile), and Very High (q=5,80−100th percentile), corresponding to increasing local tumor cell density. Zone 1 therefore represents the tumor-sparse periphery and zone 5 the tumor-dense core. By construction the zones are mutually exclusive and exhaustive: $\overset{5}{\underset{q=1}{⋃}}{ℐ}_{q}=ℐ$ and ${ℐ}_{q}\cap {ℐ}_{q^{\prime }}=\emptyset$ for $q\neq q^{\prime }$.

The quantile-based stratification is preferable to fixed-threshold partitioning because it adapts to section-level distributional shifts in tumor cell density, a well-documented feature of intratumoral heterogeneity in CRC. The use of a KDE-smoothed density surface rather than a raw per-cell count additionally captures the spatial continuity of tumor cell neighborhoods, ensuring that zone boundaries reflect mesoscale tissue architecture rather than local cell count fluctuations. These five zones are carried forward as the Q=5 study-specific partitions in the multi-study factor analytic (MSFA) layer of ISPat-3D, where each serial section of CRC1 constitutes one study.

### Cell type-specific KDE surface construction.

To obtain a continuous, spatially resolved compositional representation of the TME at each cell location, we computed cell type-specific KDE surfaces for all nine cell types across each of the 25 CRC1 sections. For each section and each cell type t∈𝒯, the XY coordinates of cells assigned to type t were used to fit a separate two-dimensional Gaussian KDE surface on a 200 × 200 grid spanning the section’s full spatial extent, using the same LOOCV bandwidth selection procedure described in Equation (17). Cell types with fewer than 30 cells in a given section were assigned a density of zero throughout that section. The fitted surfaces were bilinearly interpolated at the XY coordinates of every cell in the section, yielding a per-spatial location vector of local neighborhood densities:

(19)
$${\hat{f}}_{i}=\left({\hat{f}}_{i,\text{Tumor}},{\hat{f}}_{i,\text{CD}8\text{T}},{\hat{f}}_{i,\text{CD}4\text{T}},{\hat{f}}_{i,\text{Treg}},{\hat{f}}_{i,\text{Tcell}},{\hat{f}}_{i,\text{Bcell}},{\hat{f}}_{i,\text{Mac}},{\hat{f}}_{i,\text{Other}},{\hat{f}}_{i,\text{Stroma}}\right),$$

for spatial location i. This encodes the local cellular composition of each cell’s spatial neighborhood in a continuous and spatially smooth form, and serves as the input marker expression matrix to the ISPat-3D spatial interaction pattern analysis pipeline.

## Appendix B: BC Data Processing

### Acquisition and cell centroid extraction.

Per-section single-cell mean intensity tables were downloaded from the Zenodo repository. Cell centroids in three dimensions were extracted from the 3D segmentation mask yielding physical coordinates (X,Y,Z) in micrometres at 1μm/pixel resolution in the XY plane and 2μm per section along the Z axis. Centroid tables were joined to the mean intensity table on cell label. Marker intensity values were winsorized at the 99th percentile to mitigate the influence of imaging outliers.

### Cell type classification.

Cell types were assigned using a hierarchical threshold-based gating strategy applied to 99th-percentile normalized marker intensities, analogous to the classification framework employed for the CRC1 specimen. Twelve cell types were defined in the following order of precedence: endothelial cells (vWF/CD31 > 0.3), macrophages (CD68 > 0.3), B cells (CD20 > 0.3 and CD45 > 0.2), plasma cells (CD138 > 0.3), CD8^+^ cytotoxic T cells (CD3 > 0.3 and CD8a > 0.2), CD4^+^ helper T cells (CD3 > 0.3 and CD8a ≤ 0.2), cancer-associated fibroblasts (CAF; Collagen I > 0.3, SMA > 0.3, and panCK < 0.2), myoepithelial cells (SMA > 0.3 and CK5 > 0.3 or CK14 > 0.3), and four epithelial tumor subtypes distinguished by their cytokeratin expression profiles: HER2-positive tumor cells (panCK > 0.3 and HER2 > 0.4), basal tumor cells (panCK > 0.3 and CK5 > 0.3 or CK14 > 0.3), luminal tumor cells (panCK > 0.3 and CK7 > 0.3), and other tumor cells (panCK > 0.3). Labels were assigned in the listed order of precedence. Cells satisfying no criterion were labeled unassigned and excluded from all downstream analyses.

### Spatial zone annotation via pan-cytokeratin intensity partitioning.

Unlike the CRC1 analysis, where spatial zones were defined by a KDE-smoothed tumor cell density surface, the compact single-tissue-block geometry of the breast carcinoma dataset and the direct availability of per-cell pan-cytokeratin (panCK) intensities permit a simpler and equally principled stratification strategy. Pan-cytokeratin is a broad epithelial lineage marker that captures all cytokeratin-expressing tumor cells regardless of molecular subtype and therefore serves as a continuous proxy for local tumor epithelial burden at each cell location.

The raw panCK intensity was normalized to the 99th percentile of the global distribution, yielding a normalized score ${\tilde{\kappa }}_{i}\in [0,1]$. The five spatial zones were then defined by partitioning the global empirical distribution of $\left\{{\tilde{\kappa }}_{i}\right\}$ at its quintiles, following the same quantile-based stratification used for CRC1 in Equation (18). The five zones are labeled Very Low, Low, Intermediate, High, and Very High, corresponding to increasing local panCK expression and hence increasing local tumor epithelial density. This approach is directly comparable to the KDE-based stratification used for CRC1 in that both methods partition cells along a continuous, biologically grounded tumor burden axis into five equally populated quantile strata. The substitution of a per-cell intensity score for a KDE-smoothed density is appropriate here because each cell already carries a direct measurement of panCK expression, making the additional smoothing step unnecessary.

### Cell type-specific KDE surface construction.

To obtain a continuous, spatially resolved compositional representation of the TME at each cell location, we computed cell type-specific KDE surfaces for all 12 cell types across each of the 152 sections, following the same procedure applied to the CRC1 specimen. For each section and each cell type t∈𝒯, the XY coordinates of cells assigned to type t were used to fit a separate two-dimensional Gaussian KDE surface on a 200 × 200 grid spanning the section’s full spatial extent, using the LOOCV bandwidth selection procedure described in Equation (17). The fitted surfaces were bilinearly interpolated at the XY coordinates of every cell in the section, yielding a per-location vector of local neighborhood densities ${\hat{f}}_{i}$ for spatial location i. This encodes the local cellular composition of each cell’s spatial neighborhood in a continuous and spatially smooth form, and serves as the input marker expression matrix to the ISPat-3D pipeline.
